# Learning based prediction of cuttings concentration for enhancing hole cleaning efficiency in eccentric and deviated wells

**DOI:** 10.1038/s41598-025-15135-0

**Published:** 2025-09-23

**Authors:** Mohamed Y. Saad, Adel M. Salem, Ahmed Farid Ibrahim, Omar Mahmoud

**Affiliations:** 1 Belayim Petroleum Company (PETROBEL), Cairo, 7074 Egypt; 2 Department of Petroleum Engineering, Faculty of Petroleum and Mining Engineering, Suez University, Suez, 43512 Egypt; 3 Department of Petroleum Engineering, Faculty of Engineering and Technology, Future University in Egypt (FUE), Cairo, 11835 Egypt; 4 Department of Petroleum Engineering and Geosciences, King Fahd University of Petroleum & Minerals, Dhahran, 31261 Saudi Arabia; 5 Center for Integrative Petroleum Research, King Fahd University of Petroleum & Minerals, 31261 Dhahran, Saudi Arabia

**Keywords:** Directional drilling, Cutting concentration, Machine learning, Eccentricity, Hole cleaning, RBFN, Petrology, Engineering

## Abstract

Directional drilling often encounters challenges such as eccentric annulus conditions caused by the weight of the drill string and oscillations, compounded by gravity-induced cuttings accumulation that obstructs flow and impedes drilling processes due to inefficient hole cleaning. This study focuses on addressing these issues by developing machine learning (ML) models to predict cuttings concentration (CA) in eccentric deviated wells, aiming to enhance predictive accuracy and optimize hole-cleaning operations. The research employs multiple ML algorithms including back propagation neural network (BPNN), radial basis function network (RBFN), and support vector machine (SVM). Models are trained using comprehensive field data from six deviated wells in the Gulf of Suez, Egypt, with inputs comprising rheological properties, drilling operation parameters, cutting transport velocity ratio (V_TR_), and carrying capacity index (CCI). The models undergo rigorous validation to ensure robustness and accuracy, employing both internal validation techniques to avoid overfitting and extensive testing across varying degrees of eccentricity. The developed RBFN model demonstrated superior performance compared to existing empirical and fuzzy logic models, achieving a relation coefficient (R) of 0.993 and an average absolute error (AAE) of 1.18 at an eccentricity degree (ε) of 0.5. In further validation within neighboring test wells, the RBFN model accurately predicted CA across different eccentricities, showing high reliability with R-values of 0.984, 0.978 and 0.971 and AAE-values of 1.1, 1.4 and 1.7 for = 0, 0.4 and 0.8, respectively. Sensitivity analyses confirmed the critical influence of V_TR_ and CCI, with their impact most pronounced at the highest eccentricity tested. This study presents a significant advancement in drilling technology by integrating advanced ML methodologies to improve the monitoring and optimization of hole-cleaning efficiency in deviated wells. The novel application of these sophisticated models offers a promising solution to real-time challenges in drilling operations, enhancing efficiency and reducing operational risks associated with eccentric deviated wells. Incorporating ML models into routine drilling operations can potentially transform standard practices, making this approach a valuable asset in the field of petroleum engineering.

## Introduction

Advancements in drilling technology made it possible to drill horizontal and deviated wells with high rates of penetration. Because of their advances in having larger than ordinary vertical wells, several directional wells can be drilled from the same location to reach the reservoir in any direction, considerably lowering overall drilling costs. Increased demand for directional and horizontal drilling has led to the utilization of optimization techniques to decrease non-productive time at the rig site and thus maximize revenues^1^. Poor hole cleaning is the most prevalent issue faced in a complicated well trajectory, and it occurs mostly in the deviated and horizontal sections of oil and gas wells^2^. Although the time required to clean the wellbore is considered a non-productive time (NPT), it is considered unavoidable and necessary for effective drilling and completion operations^3^.

As the wellbore inclination angle increases, so does the eccentricity of the drill pipe. Increased eccentricity causes a reduction in fluid velocity on the annulus’s bottom side. Gravity and low fluid velocity interact to improve cutting build-up, making cutting beds easier to form^4,5^. Drill pipe is considered entirely eccentric if it lies against the inside diameter of the enclosing pipe or hole and concentric if it is exactly centered in the outer pipe or hole. The numerical findings of^6^ show that drill pipe eccentricity (ε) can enhance cuttings accumulation. The annular cutting concentration (CA) is almost the same for eccentricities of (ε= 0 and ε= 0.2). In contrast, at an eccentricity of (ε= 0.4), the annular CA is considerably increasing. As a result, hole-cleaning efficiency declines as the eccentricity of the circular drill pipe rises. For this study, several levels of presumed values of (ε= 0, 0.4, and 0.8) were considered as an explicit dependent variable in CA intelligent modeling.

Poor hole cleaning accounts for most pipe-sticking problems. Aside from pipe sticking, improper hole cleaning can cause excessive drill bit wear, reduced ROP, higher equivalent circulating density (ECD), formation fracture, lost circulation during tripping increased drag, torque forces and eventually raising drilling expenses^7,8^. Furthermore, problems with other operations such as casing and cementing activities, as well as wireline logging^9^. Hence, in order to achieve safe and effective drilling, cuttings transport characteristics and hole-cleaning efficiency must be properly understood.

From previous work, pipe hole eccentricity, drilling fluid rheology, carrying capacity index, (CA), cutting slip and transport velocities have the most substantial and direct impact on cuttings removal^10–16^. Although those parameters directly affect hole cleaning efficiency, creating such defined values for them and quantifying their interdependence on the wellbore cleanout procedure remains difficult. Drill pipe eccentricity has a negative impact on cuttings transport in inclined and horizontal wellbores^6,17^. Drill pipe eccentricity divides the flow cross-section into two regions: wide and narrow. In the large zone, the pipe causes the fluid to flow faster, whereas in the small section, it causes the fluid to flow slowly. The wall and pipe effects grow complex with drill string rotation, resulting in a decrease in flow velocity at a given pressure gradient. The fluid moves in a helical flow pattern from the narrow stationary zone to the high-speed wider region as the pipe rotates. The flow causes the fluid to experience alternate acceleration, which increases frictional pressure loss^18,19^.

Experimental studies are the most direct and precise way to determine CA parameter in the wellbore. In addition, empirical and intelligent models are constructed based on experimental data to predict hole cleaning efficiency. However, experimental studies have their limitations as it is considered a challenging task to conduct cuttings transport experiments under downhole conditions^20–22^. On the other hand, empirical and intelligent models are not based on physical laws outside the laboratory boundaries^23^.

The industry has been attempting to solve the problem of hole cleaning. Although much progress has been made, the hole cleaning process is still not fully understood and remains a key issue, particularly in inclined and horizontal wells. Over the last decade, ML approaches have found widespread use in the oil and gas sector^23–31^. These techniques have been used in reservoir engineering, drilling, and production operations which have been considered tractable, efficient, and cheap to employ. The increasing acceptance of various ML algorithms in the oil and gas industry is noteworthy^32^. It can do multi-parameter optimization with dependable robustness^33^. Table 1 summarizes some of the most implemented ML models for predicting cuttings transport efficiency.

This paper outlines a methodology for developing backpropagation neural network (BPNN), radial basis function network (RBFN), and support vector machine (SVM) models to identify the most suitable machine learning (ML) technique for predicting cuttings concentration (CA) under various eccentricity scenarios. The models were trained and tested using 4,512 raw field data records comprising eight operational and fluid parameters acquired from six deviated wells targeting an oil reservoir in the Gulf of Suez, Egypt. The performance of the proposed RBFN model was further benchmarked against three existing models: (1) an experimental setup model^34^, (2) a fuzzy logic (FL) model^23^, and (3) a generalized statistical model^35^.

Previous efforts (summarized in Table 1) predominantly relied on limited laboratory experiments or narrow simulation settings, with models often constrained by either static geometries, simplified fluid dynamics assumptions, or incomplete parameter scopes, particularly lacking in sensitivity to eccentricity levels and intermediate variables such as V_TR_ and CCI. Additionally, few studies employed robust model comparisons across multiple ML algorithms with field-scale validation. The novelty of this work lies in its integrated, field-calibrated ML framework that not only incorporates intermediate parameters derived from physical drilling conditions but also systematically assesses model performance under varied eccentric geometries. This study is the first to comprehensively compare BPNN, RBFN, and SVM against leading empirical models using real-world deviated well data, offering a more generalizable and practically applicable solution to hole cleaning prediction.

This paper is arranged as follows: Sect. 2 presents a description of the ML models implemented to estimate CA; Sect. 3 describes the methodology used to develop the radial-based, backpropagated, and support vector ML models using field observations from deviated wells at varying presumed eccentricity values; Sect. 4 presents the results and discussion on ML development and the selection of the best CA prediction model; Sect. 5 delivers a statistical comparison of CA estimates generated by ML models and existing empirical correlations; and finally, concluding remarks are offered in Sect. 6.

**Table 1:** Summary of ML studies for predicting CA and hole cleaning efficiency.

Studies	Objective	Studied parameters	Application
^36^	Estimation of the height of stationary cuttings beds deposited in horizontal and highly inclined wellbore	Pump rates, fluid density and viscosity, ROP, wellbore geometry	Artificial neural networks (ANN)
^37^	Estimation of cuttings transport in horizontal and deviated wells	ROP, flow velocity, inclination angle, pipe rotation	ANN, SVM, and K-nearest neighbors (KNN)
^38^	prediction of cuttings concentration in underbalanced drilling	ROP, flow velocity, inclination angle, pipe rotation	ANN, RBFN
^39^	Using support vector machine (SVM) technique to measure the hole cleaning efficiency in horizontal drilling	Eccentricity, ROP, flow rate, inclination angle, pipe rotation, annulus size, temperature	SVM
^40^	Modeling cuttings concentration using experimental data utilizing several machine learning (ML) techniques	Fluid density, yield point, plastic viscosity, flow rate, temperature, inclination angle, hole eccentricity, pipe rotation, ROP	Random forest, gradient boosting, adaptive boosting, stacked regression model
^23^	Estimation of downhole cuttings concentration	Annulus size, eccentricity inclination angle, ROP, cuttings density, temperature, fluid density, apparent viscosity, cuttings size, flow rate, pipe rotation	Fuzzy logic models (FL)
^41^	Predicting cuttings bed height in the well bore	Flow rate, cuttings size, ROP, eccentricity, wellbore diameter	ANN, SVM, Long Short-Term Memory (LSTM) and recurrent neural network (RNN)
^42^	Developing a data-driven model for predicting hole cleaning cuttings transport efficiency in deviated drilling	Bit type, bit drilling time, pipe rotation, weight on bit, torque, formation type, rock properties, hydraulics, drilling mud properties	Random forest, linear regression, neural networks, multivariate adaptive regression spline, support vector machine, and boosted decision tree

## Theory of ML models

### Back propagated neural network (BPNN)

ANN is the most widely used technique for regression and identifying data patterns is the. It is a type of information processing system that attempts to imitate the functionality of the human nervous system. Feed-forward back propagation neural network (BPNN) is a network that is trained using the backpropagation algorithm to establish its weights and biases. To reduce the disparity between the predicted and actual output, back propagation algorithm modifies the weights starting with the output layer and moving backward to the first hidden layer. According to^43^, a multilayer BPNN is a network with at least three layers, comprising an input layer, a hidden layer, and an output layer, with multiple neurons making up each hidden layer. where the input and output layers consist of input variables and CA as an output, respectively. The output of a BPNN with one hidden layer and one output can be expressed as follows:

1 $$\:Y=\left(\sum\:_{j=1}^{m}{\:w}_{j1}\times\:f\left(\sum\:_{i=1}^{n}{w}_{ij}\times\:{X}_{i}+{b}_{j}\right)+{b}_{1}\right),$$

where $\:{b}_{j}$ stands for the bias for the neurons in the hidden layer, $\:{b}_{1}$ for the bias for the output, $\:f$ denotes the transfer function and $\:{X}_{i}$ stands for the network input variables.

### Support vector machine (SVM)

SVMs are supervised ML models that assess data to solve classification or regression issues. SVM is a machine learning (ML) algorithm that is highly generalizable and perform well with both minor data samples and feature spaces with many dimensions^44,45^. The ability to handle regression problems has been expanded, and SVMs perform quite well in this regard^46,47^. The fundamental concept behind SVM regression is that problems are solved by mapping data into a high-dimensional feature space to find out the relationship between the input and output in the new space by creating a hyper-plane in which the data are divided into two groups. The hyperplane in the higher dimension is defined using a technique known as the kernel trick without mapping the data into a higher dimension. This results in significant computing capacity savings.

For linearly non separable data sets, kernels are employed to transfer the feature vectors from input space to kernel space, which necessitates computing power for the kernel matrix computation. The widely used kernel functions are the randomized blocks analysis of variance (ANOVA RB) kernel, the Gaussian RBF, the polynomial kernel function, and the linear kernel function as will be indicated later in applications chapter. The kind of data collection largely determines the choice of kernel functions. In comparison to the polynomial kernel, the linear kernel performs better and is beneficial for big sparse data vectors.

### Radial based network (RBFN)

RBFN is trained as same as BFNN since it shares an analogous architecture with BPNN. Specialized activation functions, such as the Gaussian function, multi-quadratics, and inverse multi-quadratics, are available for the hidden layer in RBFN. As opposed to BPNN, which creates global and unbounded activations, these activation functions have the benefit of providing localized, limited, and radially symmetric activations which reduce the distances from function centers. The Gaussian activation function used in the hidden layer is centered on a vector in feature space. From the input layer to the hidden layer, there are no weights. According to^48^, all *j-th* Gaussian hidden units get the input layer directly. The RBF output for the kth unit is presented in Eq. (2) through (4) as:

2 $$\:Y={B}_{0}+\sum\:_{j=1}^{h}{w}_{jk}\:*\:{H}_{j},$$

where $\:{w}_{jk}$ is the output layer wight and $\:{H}_{j}\:$is the *j-th* hidden unit radial basis output and is defined as:

3 $$H_{j} = f\left( {\left\| {x - C_{j} } \right\|} \right),$$

where $\:{C}_{j}$ is the center of the RBFN and ${\left\| {x - C_{i} } \right\|}$is the Euclidean distance.

The RBF of the neurons in the hidden layer was attained using a Gaussian function in the following way:

4 $$f\left( {\left\| {x - C_{i} } \right\|} \right)={e}^{\left(\frac{-{\left(\left\| {x - c_{i} } \right\|\right)}^{2}}{{{r}_{i}}^{2}}\right)},$$

where the Gaussian function is $f\left( {\left\| {x - C_{i} } \right\|} \right)$. Gradient descent learning algorithm is the most common method for updating the center$\:\:{c}_{i}$ and $\:{w}_{jk}$.

Table 2 summarizes a descriptive comparison of the three ML techniques used for CA prediction in this study.

**Table 2:** Comparison and description of artificial intelligence techniques.

Description	BPNN	RBFN	SVM	
Learning signal transmission	The information moves forward from the input neurons, through the hidden neurons to the output.	The information moves forward from the input neurons, through the hidden neurons to the output.	By performing optimal data transformations that determine boundaries between data points based on predefined classes, labels, or outputs.	
Model processing technique	This means that for training pairs (x, y), the feedforward neural networks compute a function f on an input x of fixed size such that f(x) ≈ y	The processing in two stages: the radial basis function determines the probability distribution between input parameters. Then the network learns the relationship between input and outputs.	Supervised classification algorithm where we draw a line between two different categories to differentiate between them.	
Learning parameters	Threshold values, weights, and biases	Radial basis function widths, locations and weights.	Regularization parameter (C) and a hyperparameter *(*gamma).	
Processing time	Slow	Fast	Slow	
Brief description	In BPNN, the learning and training procedure results in a gradient descent. Although the process of updating weights in multi-layered perception is almost comparable, it is more formally known as back-propagation. In these circumstances, the network hidden layers are each changed in accordance with the output values generated by the top layer.	Three-layer neural networks called radial basis function networks can represent an N-dimensional space locally. This is created by the limited influence zone of the radial basis function. A reference vector (core or prototype) and the influence field’s dimension (j) are the basis function’s input parameters. The Similarity distance between the input vector x and the prototype vector j, as well as the size of the influence field, affect the response of the radial basis functions.	Support vector machine (SVM) is a machine learning algorithm that determines boundaries between data points based on predefined classes, labels, or outputs. It uses supervised learning models to solve complex problems related to classification, regression, and outlier detection.	

## Methodology for developing ML models

### Dataset characterization

A field dataset compiled from the daily drilling reports of six offshore deviated wells oil fields is used in this study, which targets an oil resource with sandstones layered with shales in the Gulf of Suez province in Egypt. The data gathered from the whole section of six wells was utilized to predict and model CA. Table 3 summarizes collected raw data, including lithologies, mud properties, well trajectories, and drilling operation parameters.

To improve the predictive capabilities of the developed ML models, we focused on intermediate variables derived from raw input features. These intermediate variables resampling cutting transport velocity ratio (V_TR_), carrying capacity index (CCI), and equivalent circulating density (ECD) which are selected because they encapsulate complex relationships in hole cleaning system that are not captured by the raw features alone. These intermediate parameters provide a more direct measure of physical hole cleaning performance in annulus. Hence, slip velocity ($\:{\text{V}}_{\text{s}}$) is calculated for different eccentric annuli utilizing power-law fluid flow model and^49,50^ correlations for varying degrees of assumed values. The distinct $\:{\text{V}}_{\text{s}}$ derived from each eccentric profile are then used for determining intermediate variables representing hole cleaning indicators (V_TR_ and CCI), Eqs. (5) and (6).

5 $$\:{V}_{TR}=\:\frac{{V}_{T}}{\stackrel{-}{{\:V}_{an}}},\:$$

where $\:{V}_{T}$ is the cutting transport velocity (ft/min) can be implied for a given depth as:

6 $$\:{V}_{T}=\stackrel{-}{{\:V}_{an}}-{V}_{s},$$

and $\:\stackrel{-}{{\:V}_{an}}$is the mean value of $\:{V}_{an}\:$in (ft/min) across whole the well depth intervals as:

7 $$\:\stackrel{-}{{\:V}_{an}}=\frac{1}{n}\:\sum\:_{i=1}^{n}{{V}_{an}}_{i},$$

where *n* is the number of $\:{V}_{an}$ values and $\:{{V}_{an}}_{i}$ is the annular velocity for each interval in (ft/min).

API recommended practice on rheology and hydraulics, term *CCI* can be defined as:

8 $$\:CCI=\frac{\left({\rho\:}_{m}\right)\left(K\right)\left({V}_{s}\right)}{400000},$$

In addition, ECD is contributed as an intermediate variable to account for the static fluid column and friction pressure in the annulus due to cuttings as follows:

9 $$\:ECD={\:\rho\:}_{m}+\frac{\left(12\right)(\varDelta\:{P}_{C})}{\left(231\right)\left(TVD\right)},$$

where$\:{\:\rho\:}_{m}$ is the mud weight in (ppg), *TVD* is the true vertical depth in (ft) and $\:\varDelta\:{P}_{C}$ is the annular pressure loss due to cuttings in (psi), should be determined as follows:

10 $$\:\varDelta\:{P}_{C}=\left(0.025\right)\left(TVD\right)\left({\:\rho\:}_{c}-{\:\rho\:}_{m}\right)\left(CA\right),$$

where$\:{\:\rho\:}_{c}$ is the cutting density in (ppg).

To ensure data integrity prior to analysis, the duplicate elimination process was performed using Matrix Laboratory (MATLAB), version R2023b, developed by The MathWorks, Inc. (Natick, MA, USA) available at https://www.mathworks.com/products/matlab.html. Duplicate records were identified based on exact matches across key parameters, including, TVD, Weight on Bit (WOB), rate of penetration (ROP), pipe rotation (RPM), and hole inclination measurements. Using the “*unique”* function, 144 duplicate records were detected and removed from the original dataset of 4512 entries, accounting for approximately 3.2% of the total. This procedure ensured that each observation in the dataset represented a distinct drilling event, thereby preventing redundancy and potential bias in subsequent outlier detection and dimensionless analysis.

Once duplicate observations have been eliminated from input data, interquartile range technique is utilized to identify data outliers caused by vibrations in the drilling operation, geological environment, or instrument accuracy. To use this approach, first, as indicated by Eq. (5), calculate the inter-quartile range (IQR) for each characteristic using the values of the first (Q_1_) and third (Q_3_) quartiles. Next, we use Eqs. (6) and (7) to find each feature’s lower inner boundary (LIB) and upper inner boundary (UIB) values. Then, data points that have values below or over LIB are regarded as outliers and are to be substituted with the median value of pertinent data series. Following the data cleaning process using the IQR approach mentioned above, around 186 data outliers were replaced, making up approximately 4.25% of post-duplicate dataset that was evaluated for CA modeling. The total percentage cleaned from original dataset is around 7.3%.

11 $$\:IQR=1.5\:\times\:\left({Q}_{3}-{Q}_{1}\right),$$

12 $$\:LIB={Q}_{1}-IQR,$$

13 $$\:UIB={Q}_{3}+IQR,$$

After careful data filtering, the inputs were normalized using Min-Max normalization technique with the normalized inputs ranges from 0 to 1 using Eq. (8)^51^:

14 $$\:{X}_{n}=\frac{X-{X}_{min}}{{X}_{max}-{X}_{min}},$$

where $\:{\text{X}}_{\text{n}}$ is the normalized value of X and ($\:{\text{X}}_{\text{m}\text{i}\text{n}}$, $\:{\text{X}}_{\text{m}\text{a}\text{x}}$) is the minimum and maximum values of the value of X, respectively.

Randomly, the field dataset for CA modeling comprises 4,512 data points and is split into three divisions. The first dataset is the training dataset represents (70%) of the whole data used in training and computing the weights and biases. The second is the validation dataset (15%). The validation error decreases normally during the training phase and increases when the network begins to be overfit. The last data set is the test set (15%) used to evaluate the network. Noting that, the numbers (70, 15, 15%) are approximate numbers.

**Fig. 1:**
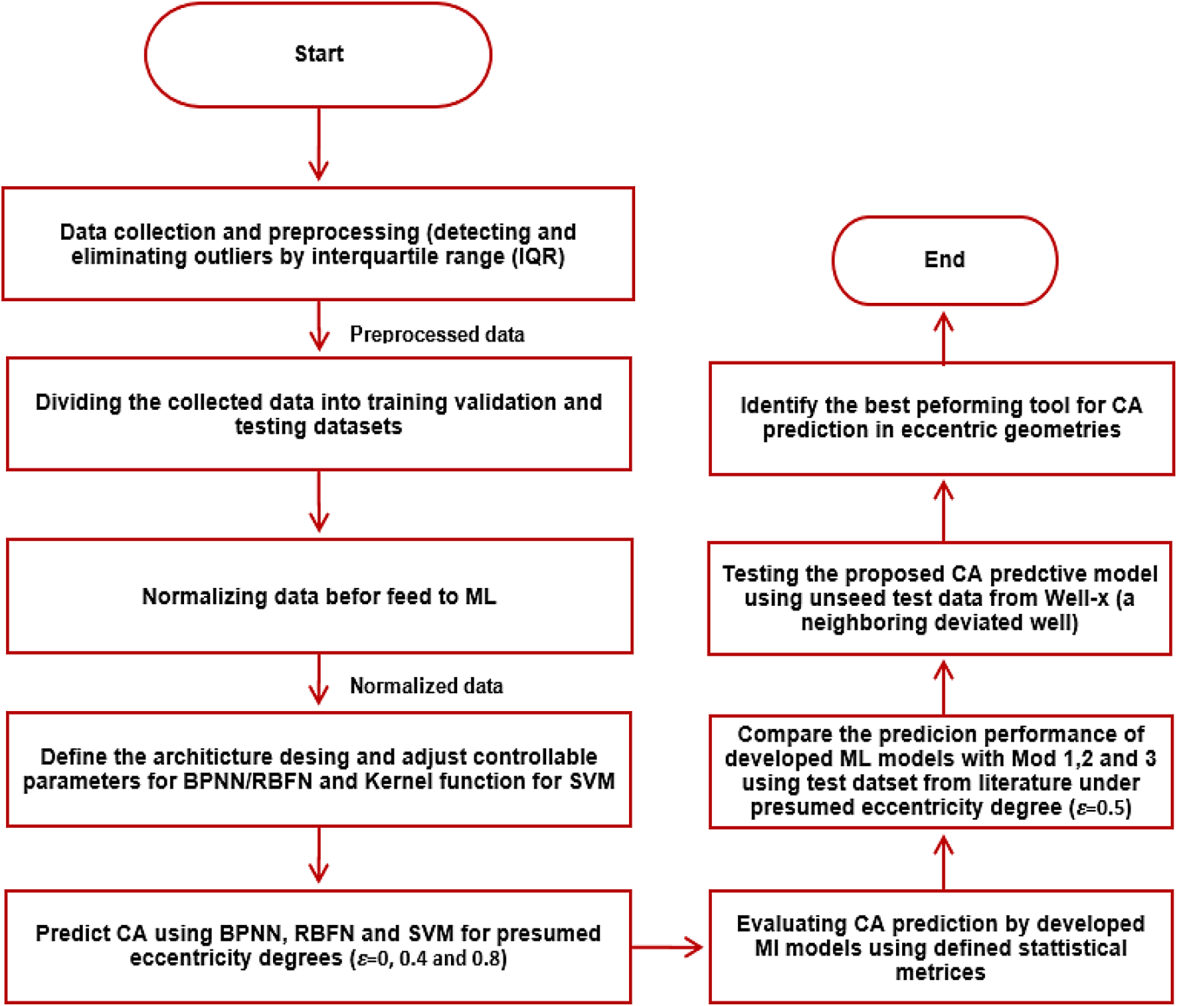
Schematic workflow diagram for CA prediction in eccentric and deviated geometries using ML algorithms.

The following methodology was used to develop and select the most suitable and efficient ML model for the current work. This model will be utilized for comparison with the two empirical models previously mentioned. Figure 1 displays a schematic workflow diagram used to predict CA using proposed ML algorithms while drilling deviated and eccentric wells.

**Table 3:** Drilling parameters, mud characteristics, lithologies, and well trajectories for the six wells.

Gathered field data	Well 1	Well 2	Well 3	Well 4	Well 5	Well 6
Measured depth (ft)	3111-12,097	503 − 11,562	400 − 11,353	2947-13,806	3316-10,095	1506-13,800
Inclination (º)	21.3–62	0.0-41.2	0.4–48	12.8–48.6	13.0-25.4	21.8–54.3
Lithology	Lime/ Salt/ Shale/ Sandstone/ Anhydrite	Lime/ Salt/ Shale/ Anhydrite	Lime/ Salt/ Shale/ Sandstone/ Anhydrite	Lime/ Salt/ Shale/ Sandstone/ Anhydrite	Salt/ Shale/ Sandstone/ Anhydrite	Lime/ Salt/ Shale/ Sandstone/ Anhydrite/ Gypsum
Mud weight (ppg)	9.2–12.8	8.5–13.8	8.8–12.0	9.6–12.6	9.9–14.5	9.5–13.5
Plastic viscosity (cp.)	25–39	10–48	7–38	16–43	14–35	19–39
Yield point (lbf/100 ft^2^)	17–42	18–48	19–36	20–43	18–42	17–55
WOB (1000 lbf)	3.9–39.1	2.53–44.9	22.5–36.3	6.2–41.1	6.9–47.7	3.06–42.92
Mud flow rate (gpm)	350–741	175–702	298–900	460–757	497–717	390-887.2
RS-motor RPM	90–119	0-124	0-135	0-114	0-113	0-136
Cuttings density (ppg)	17.9–24.9	17.9–24.9	17.9–24.9	17.9–24.9	17.9–24.9	17.9–24.9
RPM	90–240	0–32	0-146	0-146	50–149	0-140
TORQ (1000 ft.lbf)	6.53–16.59	0-20.5	0-13.4	0-18.8	5.5–18.7	0-26.28
Stand pipe press (psi)	946-2,983	654-3,840	500-2,400	1654-3,818	1810-3,334	908-3,016

### Developing back propagation neural model (BPNN)

**Fig. 2:**
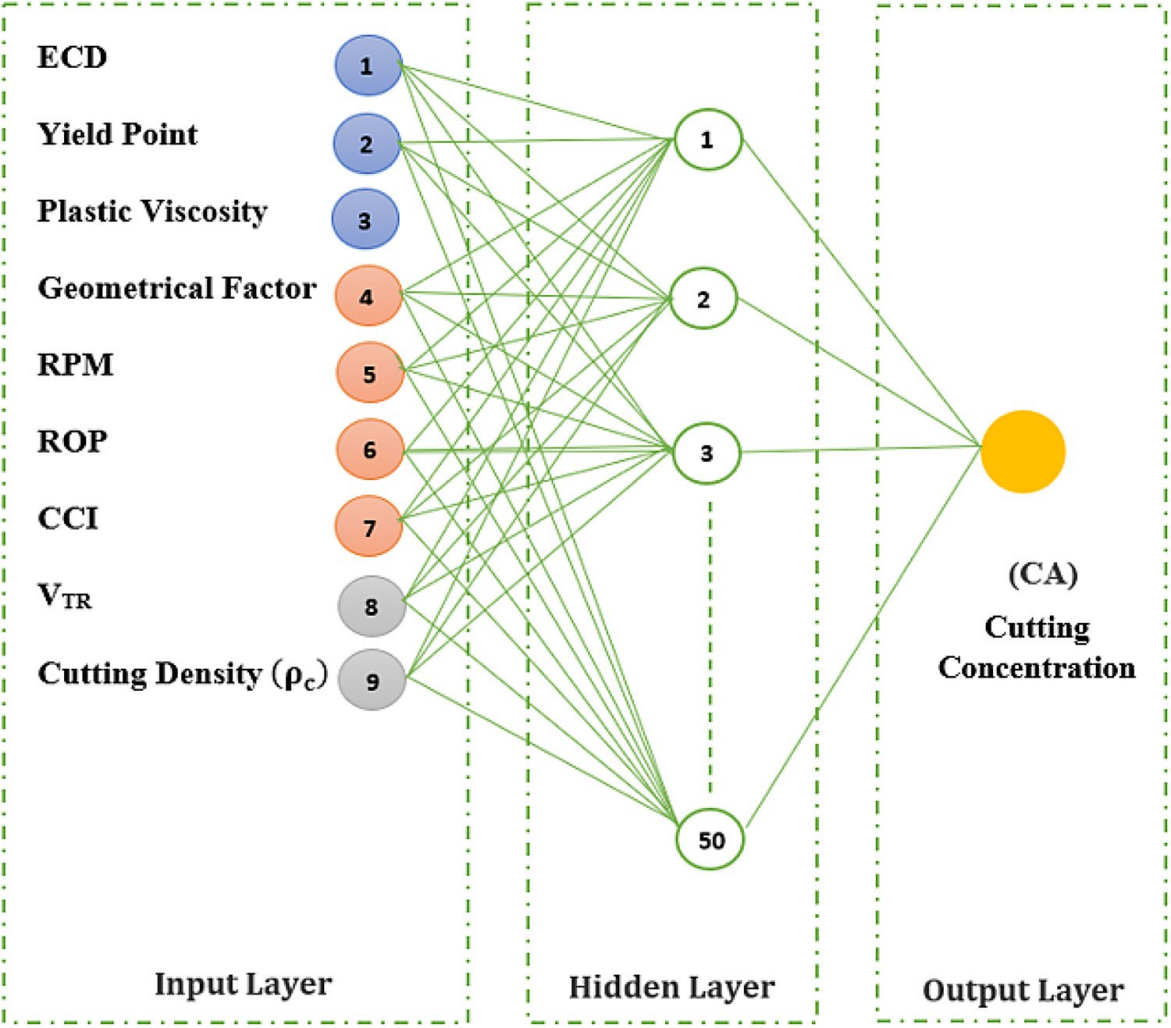
Schematic diagram depicting a single-hidden-layer BPNN model with 50 neurons, 9 inputs and one single (CA) output.

In this NN technique, feed forward Back propagation learning algorithm was applied with several runs to realize the sensitivity for input parameters for each pipe hole eccentricity^52^. The number of hidden layers, their neurons, weights and biases values will be on iterative mode using the MATLAB software by implementing different transfer functions to achieve the best correlation coefficient (R). As illustrated in Fig. 2, the neural network design has three layers: (1) an input layer with nine input neurons of (ECD), yield point (Yp), plastic viscosity (Pv), hole geometrical factor (G), RPM, ROP, CCI, V_TR_, cutting density ($\:{{\uprho\:}}_{\text{c}}$); (2) a hidden layer with 50 processing neurons; (3) an output layer with one output neuron for cutting concentration CA as a hole cleaning efficiency indicator. For developed BPNN, log-sigmoidal and radial basis transfer functions have been used.

The following elements should be established while developing an ANN: (1) network architecture; 2) training algorithm; 3) activation function. The conventional neural network for CA prediction uses a feed-forward BPNN architecture with three layers: input, hidden, and output layers. Gradient descent, and Levenberg-Marquardt are the two algorithms that have been widely used for training or optimizing ANNs for ROP prediction because of their simplicity. Numerous studies have employed these techniques to enhance neural networks for prediction, improving prediction accuracy^53–55^. Additionally, the aforementioned studies on ROP modelling using BPNN utilized only one hidden layer as the most preferred technique because of its straightforward and accurate performance as well as to avoid overfitting by adding more layers^56,57^. For these reasons, just one hidden layer was used in this thesis.

**Fig. 3:**
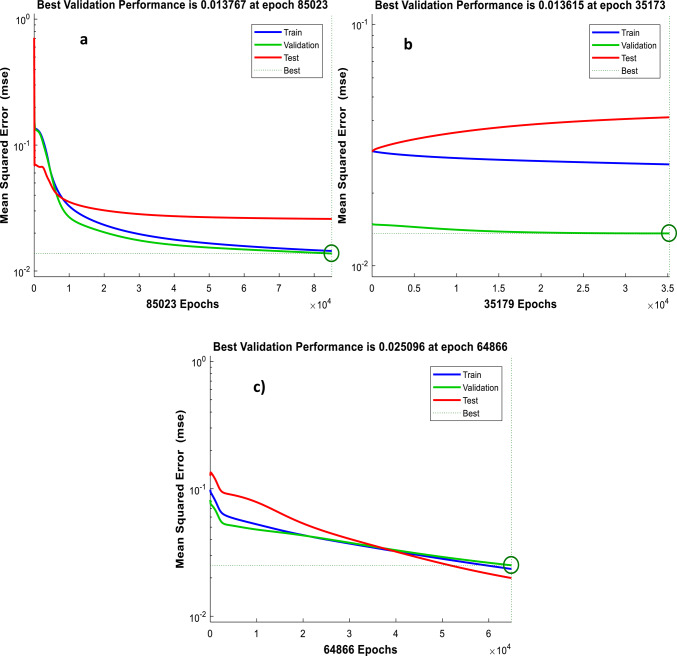
Performance curve of the BPNN modelling: (**a**) ε=0; (**b**) ε=0.4; (**c**) ε=0.8.

**Fig. 4:**
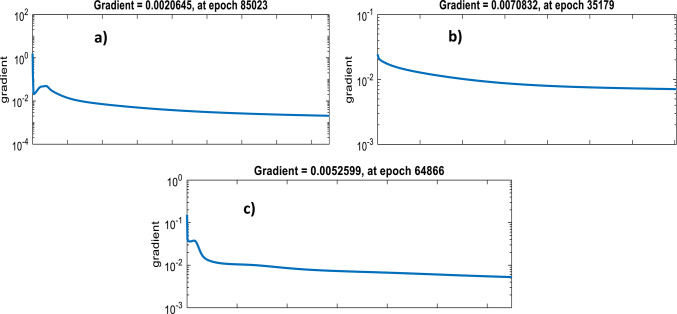
Training plots for: (**a**) ε=0; (**b**) ε=0.4; (**c**) ε=0.8 BPNN modelling.

As depicted from performance plots (Fig. 3), when the error in validation set reaches over iterations the training process stops and the minimum error has been recorded at the end of 85,023, 35,173 and 64,866 iterations for (=0, =0.4 and =0.8) BPNN modelling, respectively as marked by the perpendicular dotted lines and the circle in the performance graph. The training gradient curves in Fig. 4 also indicates that the network shows no learning progress over iterations for =0 BPNN modelling, same as =0.4 and =0.8 modelling.

### Developing radial based model (RBFN)

**Fig. 5:**
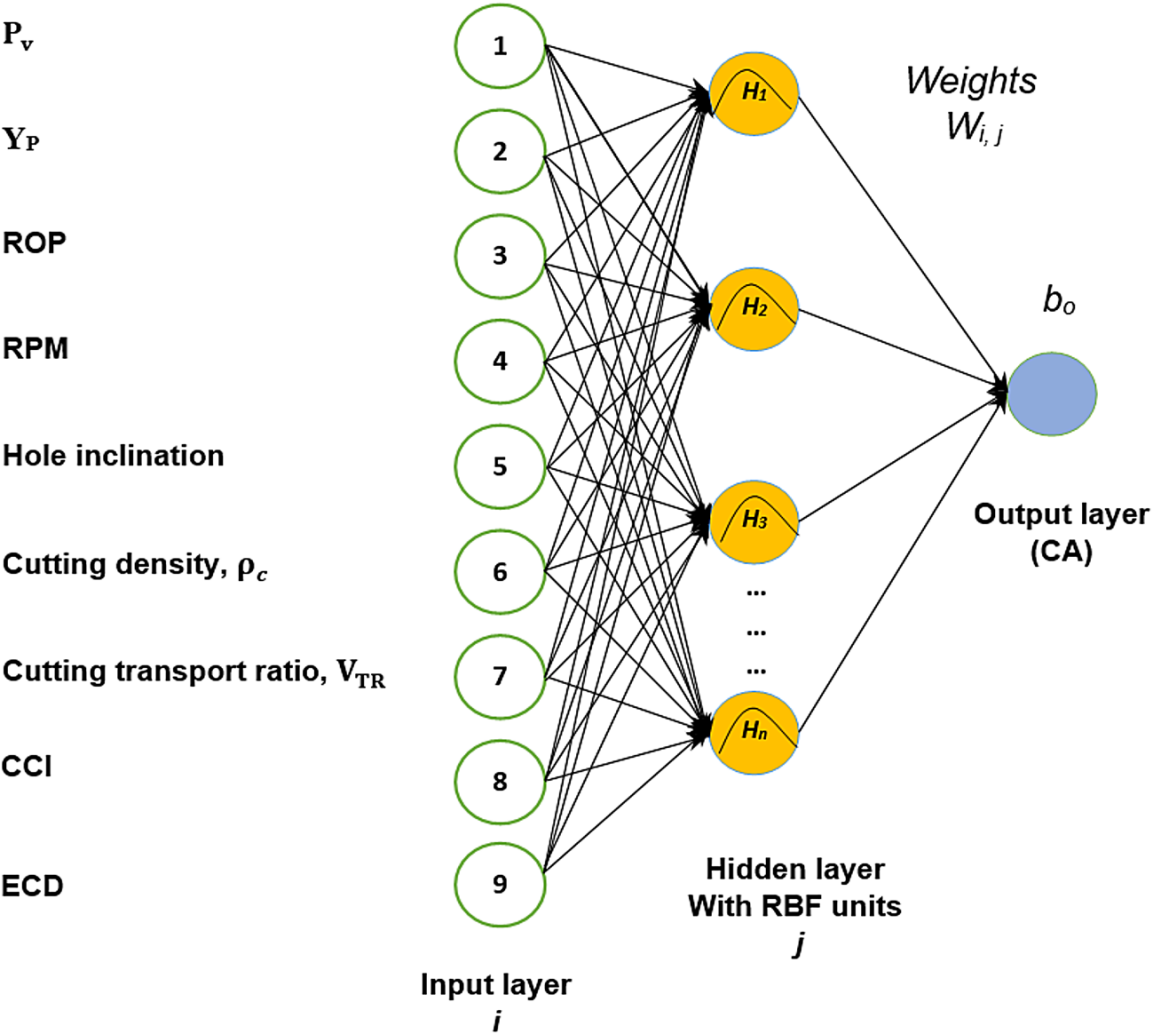
Architecture of RBFN for CA prediction including intermediate parameters (V_TR_, CCI and ECD).

In this work, the RBFN algorithm is designed using RBF neural network code in MATLAB. Regarding sensitivity analysis, the spread constant value has been changed until reaching maximum R with input parameters the same as the sensitive BPNN input number to ensure the best-fitted correlation and lowest average error. The dataset entry design is the same as the BPNN training, validating, and testing sets. Figure 5 shows the architecture of RBFN used for CA prediction.

To mitigate overfitting and improve generalization in the Radial Basis Function (RBF) neural network, we applied a combination of regularization and validation-based model selection. The RBF model was implemented using “*newrb*” function Eq. (9) with a spread parameter set to 0.6 to control the smoothness of the radial basis functions. A smaller spread emphasizes local responses and increases sensitivity to the input space, while still offering regularization. To ensure that the full specified network structure was utilized, the mean squared error (MSE) goal was set to zero, preventing early stopping and enabling explicit control over model complexity. The dataset was manually split into training (70%), validation (15%), and testing (15%) subsets.

15 $$\:Network\:=\:newrb\:(p,\:t,\:goal,\:spread,\:mn,\:df),$$

where *p*,* t* are the input and the target, the goal for mean squared error is set to be 0.005, $\:mn$ stands for maximum neurons and $\:df$ is the number of neurons to add in between displays.

**Fig. 6:**
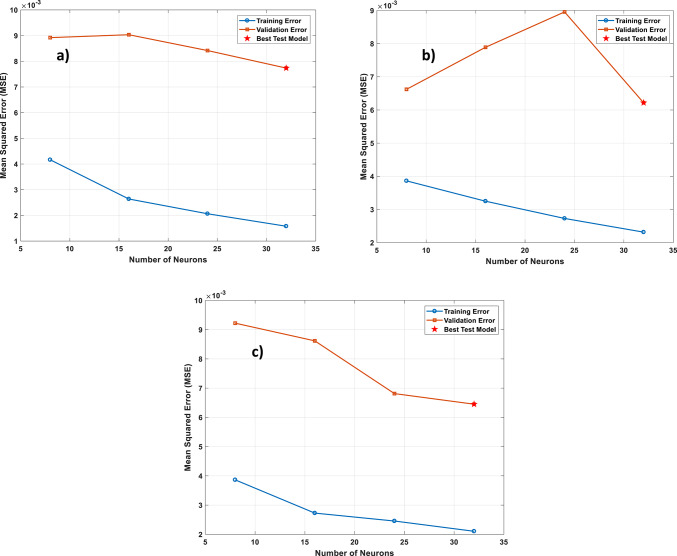
Training and validation learning curves of the RBFN test modelling: (**a**) ε=0; (**b**) ε=0.4; (**c**) ε=0.8.

RBFN was trained using varying neuron counts from 8 to 32 in $\:df$ steps of 8. At each eccentricity level, training and validation MSEs were recorded and plotted as learning curves. The optimal model was chosen based on the minimum validation error, balancing model complexity and generalization. The selected model was finally evaluated on a separate test set to assess its performance on unseen data. For each network configuration, training and validation MSE were recorded. These values were plotted to generate learning curves shown in (Fig. 6), providing insight into model behavior as complexity increased. The optimal number of neurons was selected based on the lowest validation error, representing the best trade-off between underfitting and overfitting.

To rigorously evaluate the generalization performance of the RBFN, a 5-fold cross-validation (CV) strategy was employed. The dataset, consisting of normalized input–output pairs derived from six wells within a single geological basin, was randomly divided into five equally sized folds. In each CV iteration, four folds (80% of the data) were used for model training, while the remaining fold (20%) served as the validation set. This process was repeated five times to ensure that each data subset was used exactly once for validation, thereby minimizing selection bias and enhancing the reliability of performance estimates.

Due to the limited heterogeneity of the dataset and the risk of overfitting, model training within each fold incorporated a moderately relaxed convergence goal of 0.01 and an upper limit of 100 neurons. This configuration was chosen to strike a balance between fitting accuracy and model complexity, promoting smoother generalization without sacrificing predictive capability.

### Developing support vector model (SVM)

In this work, the ANOVA RBF kernel is used for regression analysis. The SVM data used in this study for hole cleaning efficiency regression and classification use the same input parameters of BPNN and RBFN, still targeting the best-fitted correlation coefficient and lowest error.

Several SVM algorithms of the linear, quadratic, cubic, and Gaussian regression models have been developed to model CA for the same different presumed eccentricities (=0, =0.4, and =0.8). The regularization parameter and the kernel function used heavily influence the performance of the SVM model. Therefore, choosing the right kernel function is essential for optimized SVM models. The connections and distributions of dataset variables affect the effectiveness of kernel functions, and there is no designated method for choosing which kernel function to use^58^. Thus, three widely used kernel functions were chosen to perform a trial-and-error analysis for SVM in this work (Table 4).

**Table 4:** Kernel functions commonly used in SVM prediction applications.

Kernel function	Equation	Descriptions of parameters
Radial basis function (RBF-kernel) or Gaussian function	$\:k\left(x,y\right)=exp\left( { - \frac{{\left\| {x - y} \right\|}}{{2\sigma ^{2} }}} \right)$	$\:{\sigma\:}^{2}$ denotes the variance of the Gaussian kernel.
Quadratic (polynomial) kernel function	$\:k\left(x,{x}_{i}\right)={\left(t+\frac{{{x}_{i}}^{T}x}{c}\right)}^{d=2}$	d and i are the degree and intercept, respectively. Hence, d = 2.
Cubic kernel function	$\:k\left(x,{x}_{i}\right)={\left(t+\frac{{{x}_{i}}^{T}x}{c}\right)}^{d=3}$	

The three previously mentioned kernel algorithms converge easier to optimal solutions when the best values of the hyperparameters obtained by the conjugate gradient (CG) method are introduced to the optimization algorithms as the current best solution. Optimization algorithms adjust the initial solutions they assign particularly depending on their own processes, based on the current best solution. The optimization algorithms make these kinds of processing over a series of iterations to minimize root mean squared error (RMSE) which could be computed by comparing the values of the target/dependent variable with the predicted value. Follows, through assessing the cost function reached by each member of a population, the optimal solution is chosen for each iteration of the optimizer. The repetition process continues until the termination condition is met, usually after a certain number of iterations. The train and test sets of the SVM model have been assigned by choosing the optimal hyperparameter values obtained in the previous iteration.

## Results and discussion

### BPNN model results

The optimal number of neurons for the developed BPNN model was 50, the best training function was the Levenberg-Marquardt, and the best transfer function was Tan-sigmoidal. The learning rate and the momentum used for the BPNN model were 10^− 4^ and 0.9, respectively. Evaluation matrices for training, validating and testing phases were recorded during the running process of BPNN model, and the parameters that performed better on the basis of relation coefficient (R) and average absolute error (AAE) considered the models optimum parameters as:

16 $$\:R=\sqrt{\frac{\sum\:_{i=1}^{n}{\left({CA}_{pred,i}-{\overline{CA}}_{act}\right)}^{2}}{{\left({CA}_{act,i}-{\stackrel{-}{CA}}_{act}\right)}^{2}}},$$

17 $$\:AAE=\frac{1}{n}\left(\sum\:_{i=1}^{n}\left|{CA}_{act,i}-{CA}_{pred,i}\right|\right)/\left(\frac{1}{n}\sum\:_{i=1}^{n}{CA}_{act,i}\right),$$

**Fig. 7:**
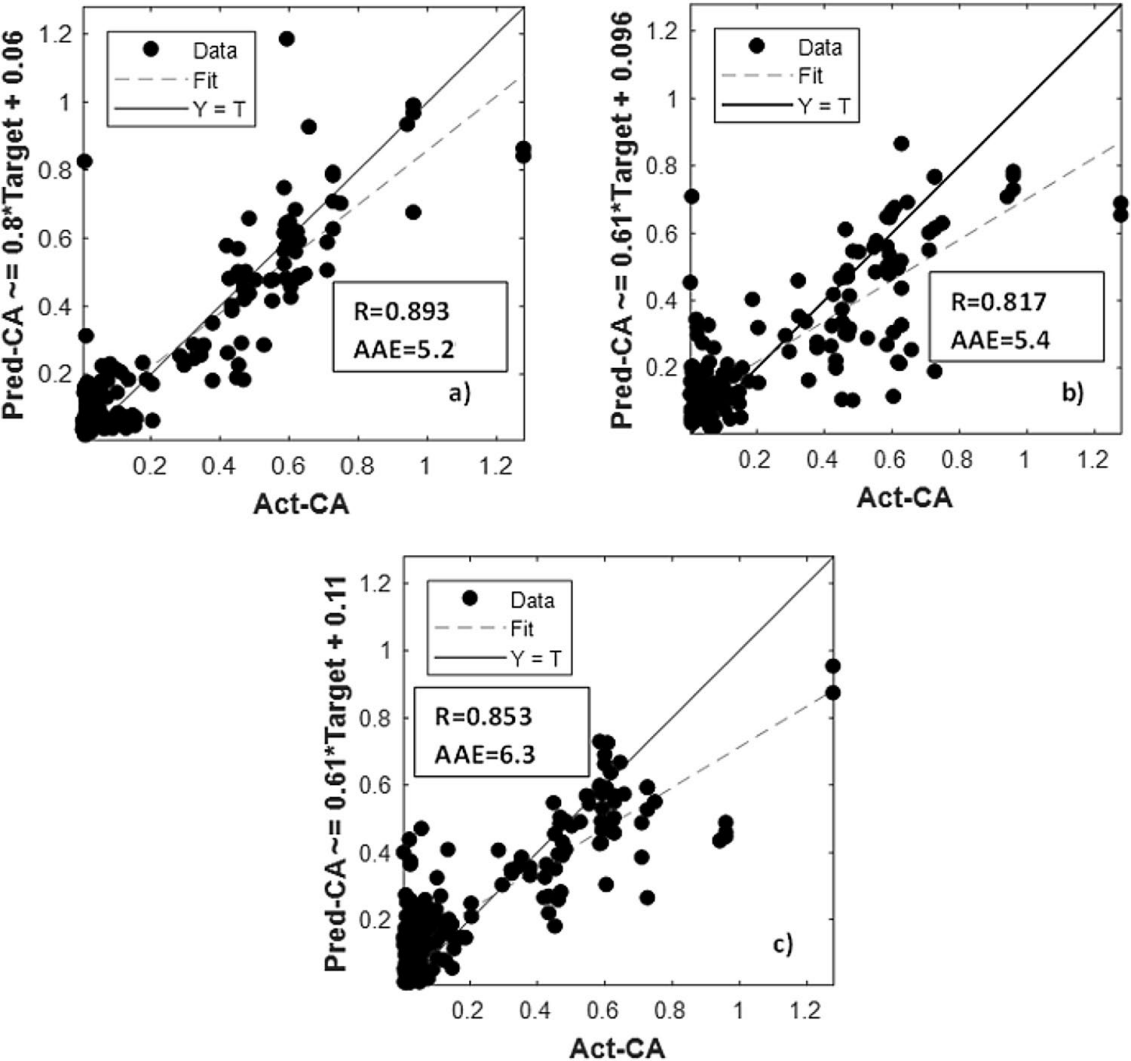
BPNN regression plots for: (**a**) ε=0; (**b**) ε=0.4; (**c**) ε=0.8 BPNN modelling.

Figure 7 demonstrates the BPNN algorithm limited capability in predicting CA, with R-values of 0.893, 0.817, and 0.853 and AAE-values of 5.2, 5.4 and 6.3 for = 0, 0.4 and 0.8, respectively. These results indicate that the developed BPNN model struggles to accurately predict CA, as evidenced by poor statistical performance metrics.

### SVM model results

**Fig. 8:**
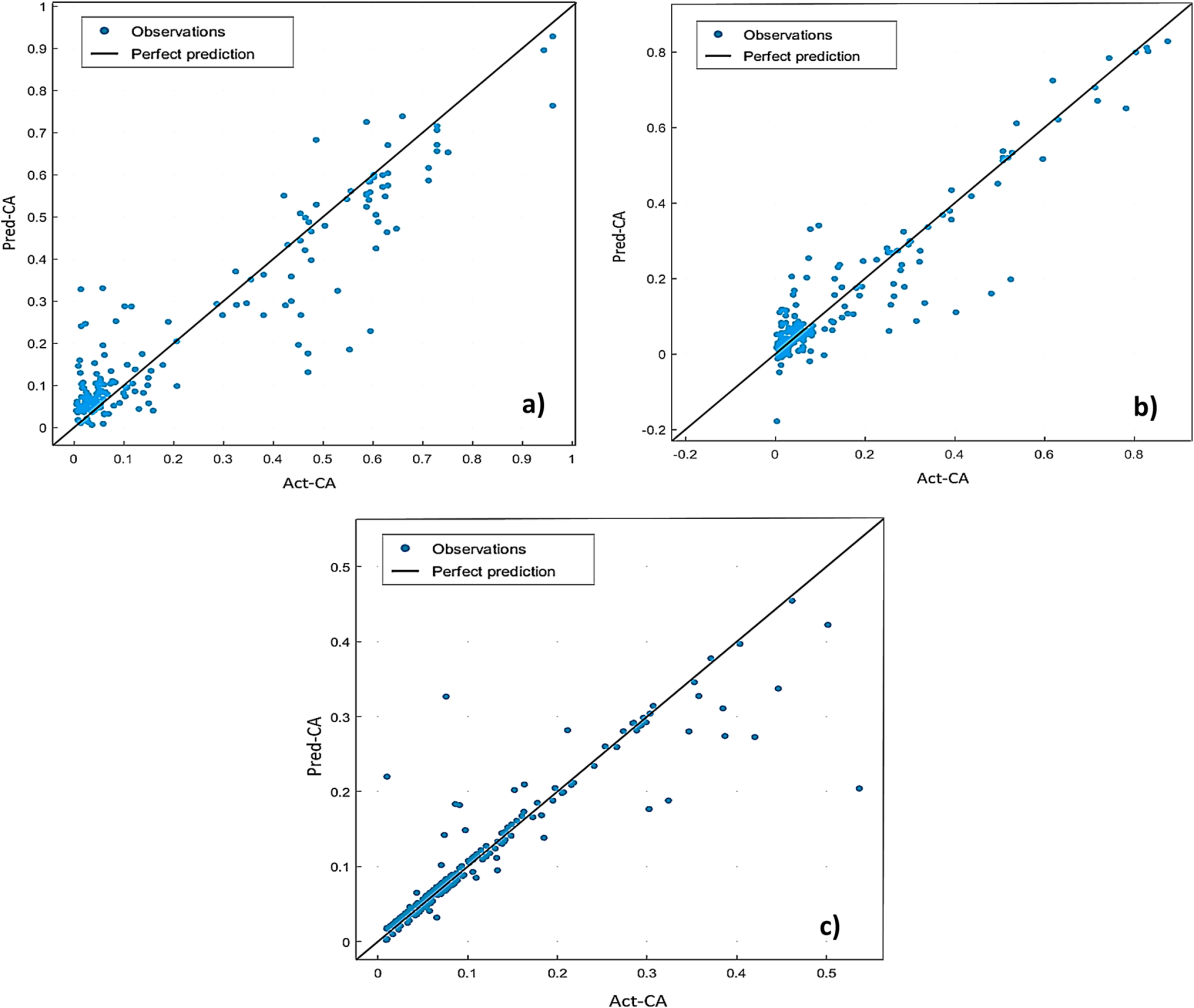
Quadratic SVM regression plots for: (**a**) ε=0; (**b**) ε=0.4; (**c**) ε=0.8 SVM modelling.

**Fig. 9:**
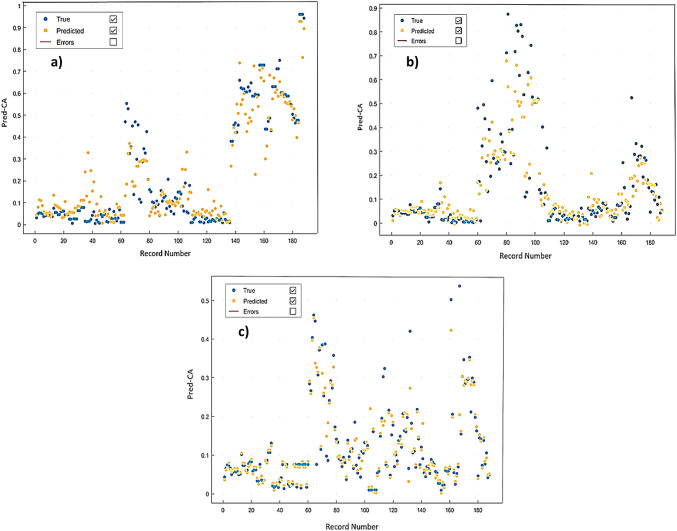
Cubic SVM response for: (**a**) ε=0; (**b**) ε=0.4; (**c**) ε=0.8 SVM modelling.

The SVM method has been used to predict CA. The results of the training show that the SVM performance model is acceptable regarding an overall R-values of (0.87, 0.86 and 0.83) and AAE-values of 4, 7 and 2 for = 0, 0.4 and 0.8, respectively. Table 5 summarizes the training results obtained for implementing SVM for the different eccentricity models, aiming for the best training correlation coefficients. Accordingly, the quadratic SVM shows the lowest AAE among SVM algorithms. Figure 8 shows the regression plots for the implemented SVM algorithms. Additionally, Fig. 9 describes the SVM response plots of predicted ad actual CA for every training record number. SVM regression results show that the best correlation coefficients are related to medium Gaussian quadratic and cubic SVM algorithms.

**Table 5:** Training results of SVM models for =0, 0.4 and 0.8.

	ε =0	ε =0.4	
	Quadratic SVM for ε=0	Medium Gaussian SVM ε=0.4	Quadratic SVM for ε=0.4
Metric results			
RMSE	0.07	0.1	0.08
R-Squared	0.871	0.862	0.833
MSE	0.01	0.01	0.01
MAE	0.04	0.07	0.05
Prediction speed	~ 1600 obs/sec	~ 12,000 obs/sec	~ 1600 obs/sec
Training time	1.5234 s	0.14319 s	0.85989 s
Model type			
Preset	Quadratic SVM	Medium Gaussian SVM	Quadratic SVM
Kernel function	Quadratic	Gaussian	Quadratic
Kernel scale	Automatic	3	Automatic
Box constraint	Automatic	Automatic	Automatic
Epsilon	Automatic	Automatic	Automatic
Features used	All features used in the model, before PCA	All features used in the model, before PCA	All features used in the model, before PCA
PCA	Disabled	Disabled	Disabled
=0.8			
Cubic SVM for =0.8			
Metric results			
RMSE		0.04	
R-Squared		0.834	
MSE		0	
MAE		0.02	
Prediction speed		~ 54,000 obs/sec	
Training time		0.093452 s	
Model type			
Preset		Cubic SVM	
Kernel function		Cubic	
Kernel scale		Automatic	
Box constraint		Automatic	
Epsilon		Automatic	
Features used		All features used in the model, before PCA	
PCA		Disabled	

### RBFN models results

RBF models adopted in this work had three layers: an input layer, a nonlinear hidden layer, and a linear output layer. The RBF network is used to develop predictions once it has been trained and its centers, radii, and weights have been specified. The RBF of the hidden layer neurons was implemented as a Gaussian function as:

18 $$\:{\varnothing\:}_{i}\left({\left\| {x - C_{i} } \right\|}\right)={e}^{-\left(\frac{-{\left({\left\| {x - C_{i} } \right\|}\right)}^{2}}{{{r}_{i}}^{2}}\right)},$$

where $\:{\varnothing\:}_{i}$ is the basis function *i*^*th*^ hidden neuron, $\:{r}_{i}$ is the RBF radii, $\:{c}_{i}$ centers of the network and $\:‖x-{c}_{i}‖\:$is the Euclidean distance. The structure of derivable purlin nonlinear function $\:y$ for the output layer with respect to the weights $\:{w}_{i}$ can be formulated as:

19 $$\:y=\sum\:_{i=1}^{m}{w}_{i}{\varnothing\:}_{i}\left({\left\| {x - C_{i} } \right\|}\right)+b,$$

where $\:{w}_{i}$ are the synaptic weights linking the hidden layer to output neurons, b is the output neurons bias term. The cosine $\:{\varnothing\:}_{i1}\left(x.{x}_{i}\right)$ and Euclidean $\:{\varnothing\:}_{i2}\left({\left\| {x - x_{i} } \right\|}\right)$ distances have been proposed to be combined using a new kernel as follows:

20 $$\:{\varnothing\:}_{i}\left(x,{x}_{i}\right)={{\alpha\:}_{1}\varnothing\:}_{i1}\left(x.{x}_{i}\right)+{{\alpha\:}_{2}\varnothing\:}_{i2}\left({\left\| {x - x_{i} } \right\|}\right),$$

where $\:{\alpha\:}_{1}$ and $\:{\alpha\:}_{2}$ are the fusion weights. At the n^th^ learning iteration associated with a particular epoch. The overall mapping only considering Euclidean $\:{\varnothing\:}_{i}\left({\left\| {x - C_{i} } \right\|}\right)$ distances can be expressed as follows:

21 $$\:{y}_{n}=\sum\:_{i=1}^{m}{w}_{i,n}{\varnothing\:}_{i}\left({\left\| {x - C_{i} } \right\|}\right)+{b}_{n},$$

**Fig. 10:**
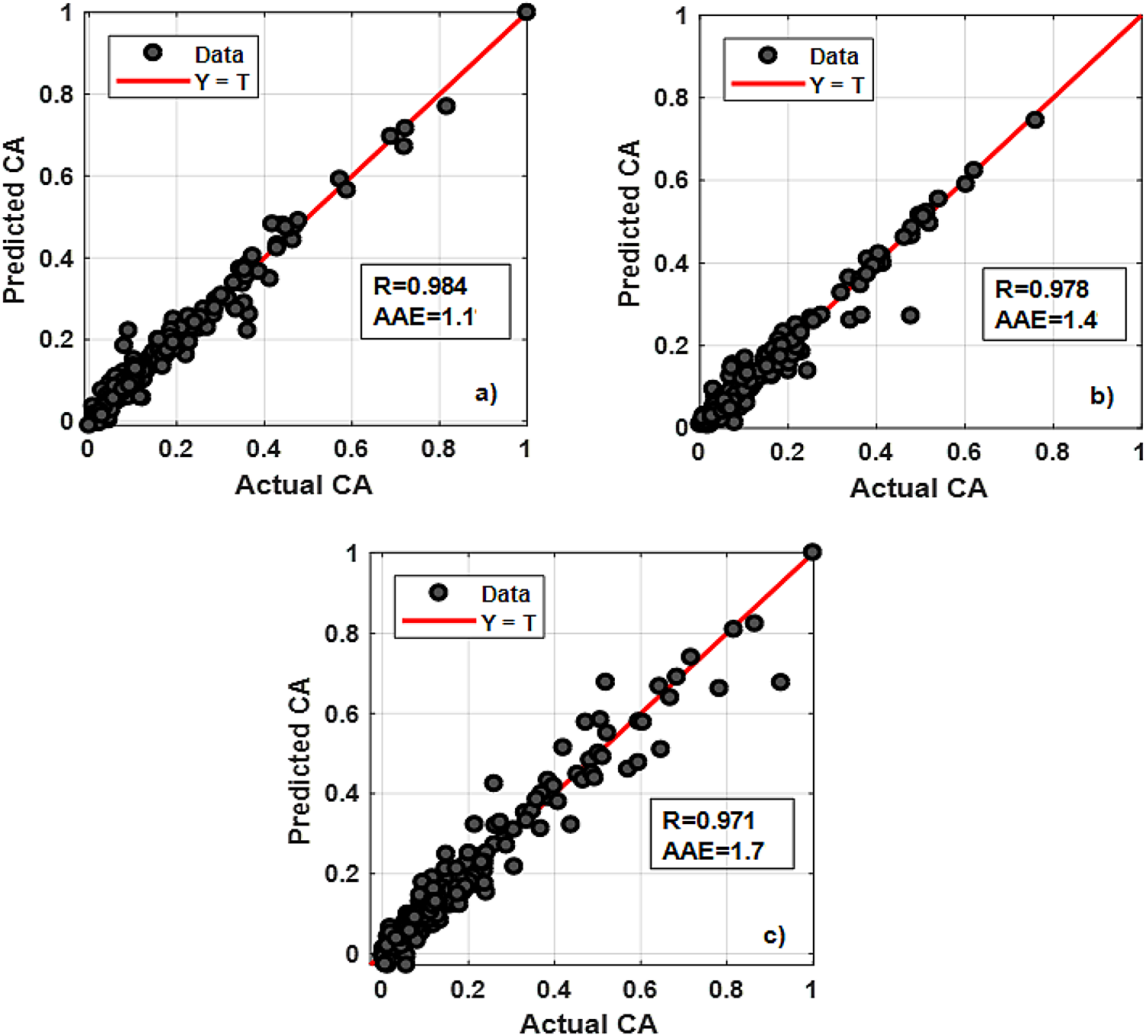
Regression plots for: (**a**) ε=0; (**b**) ε=0.4; (**c**) ε=0.8 RBFN modelling.

Figure 10 shows the regression plots for different eccentricity levels ε=0, 0.4, and 0.8, giving overall relation coefficients R-values of 0.984, 0.978, and 0.971 and average absolute errors AAE-values of 1.1, 1.4 and 1.7, respectively.

Furthermore, to comprehensively assess the performance and generalization capability of the RBFN model, additional statistical metrics were calculated, including root mean squared error (RMSE), mean absolute percentage error (MAPE), the coefficient of determination (R^2^), and adjusted R^2^. These metrics were computed across the training, validation, testing, and combined datasets using the denormalized predicted values as in Eqs. (16) through (19).

22 $$\:MSE\:=\left(\frac{1}{n}\right){\sum\:}_{i=1}^{n}{\left({CA}_{pred,i}-\:{CA}_{act,i}\right)}^{2},\:$$

23 $$\:MAPE\:=\:\left(\frac{100\%}{n}\right){\sum\:}_{i=1}^{n}\left|\frac{{CA}_{pred,i}-\:{CA}_{act,i}}{{CA}_{act,i}}\right|,$$

24 $$\:{R}^{2}=\:1\:-\frac{\:{\sum\:}_{i=1}^{n}{\left({CA}_{act,i}-\:{CA}_{pred,i}\right)}^{2}}{{\sum\:}_{i=1}^{n}{\left({CA}_{act,i}-\:{\overline{CA}}_{act}\right)}^{2}},$$

25 $$\:Adjusted\:{R}^{2}=\:1\:-\:\left(1\:-\:{R}^{2}\right)*\:\left(\frac{n\:-\:1}{n\:-\:p\:-\:1}\right),$$

where $\:{CA}_{pred,i}$ is the predicted value of $\:CA$, $\:{CA}_{act,i}$ is the actual value of $\:CA$, is the number of samples and $\:p$ is the number of predictors (features).

These statistical metrics were computed across the training, validation, testing, and combined datasets using the denormalized predicted values for different eccentricity values ( ε= 0, 0.4, and 0.8) as listed in (Tables 6, 7 and 8).

**Table 6:** Results of CA prediction using RBFN for (ε=0).

	ε=0							
	*R*	*R* ^2^		Adjusted. *R*^2^		AAE	RMSE	MAPE (%)
Training	0.987	0.974		0.974		1.2	0.048	3.9
Testing	0.977	0.955		0.952		1.4	0.094	5.8
Validating	0.963	0.927		0.923		1.1	0.089	5.9
Overall	0.984	0.968		0.966		1.2	0.062	6.1

**Table 7:** Results of CA prediction using RBFN for (ε=0.4).

	ε=0.4							
	*R*	*R* ^2^		Adjusted. *R*^2^		AAE	RMSE	MAPE (%)
Training	0.974	0.949		0.945		1.5	0.078	4.8
Testing	0.923	0.852		0.844		1.7	0.176	5.2
Validating	0.981	0.962		0.959		1.9	0.048	6.4
Overall	0.978	0.957		0.953		1.8	0.074	7.1

**Table 8:** Results of CA prediction using RBFN for (ε=0.8).

	ε=0.8							
	*R*	*R* ^2^		Adjusted. *R*^2^		AAE	RMSE	MAPE (%)
Training	0.983	0.966		0.963		1.9	0.089	5.6
Testing	0.941	0.886		0.876		2.1	0.192	8.1
Validating	0.953	0.908		0.899		1.9	0.093	7.3
Overall	0.971	0.943		0.938		2.1	0.046	7.8

### Validation of proposed RBFN model

**Fig. 11:**
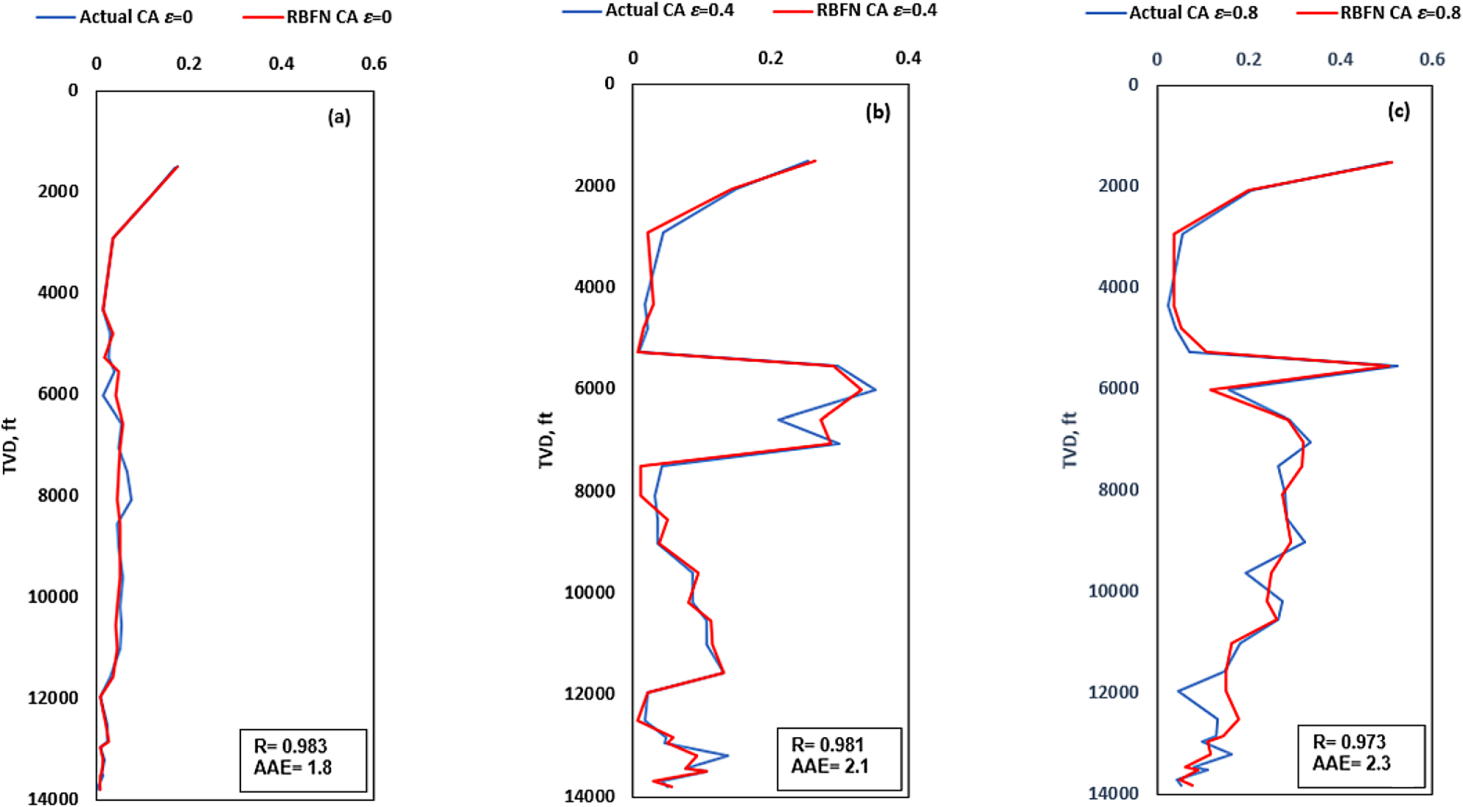
CA prediction using RBFN for neighbor test well application (0 to14000) ft depth interval: (**a**) CAε=0; (**b**) CAε=0.4; (**c**) CAε=0.8.

To validate the proposed RBFN model, it was used to predict CA in a real-world scenario while drilling new deviated test well located in the Gulf of Suez, Egypt. An overview of the lithological remarks, depth intervals, and actual hole cleaning indicators for well-x, are indicated in (Table 9). In addition, Fig. 11 depicts the utilization of RBFN model for the new validation well-x datapoints showing a high CA prediction accuracy for R-values = 0.987, 0.977 and 0.968 and AAE-values of 0.8, 1.97 and 1.6 for ε= 0, 0.4 and 0.8, respectively.

**Table 9:** Summary drilling and lithological parameters for validation with well-x (1506 to 13800) ft.

DO, in	DH, in	TVD, ft	Lithology remarks	$\:{\mathbf{V}}_{\mathbf{T}\mathbf{R}}$	CCI	CA	ECD, ppg
26	6.75	1506	Gypsum	0.265	0.095	0.254	12.809
		2068	Clay	0.478	0.26	0.149	11.071
		2908	Salt	0.594	0.4	0.043	10.388
		4325	Anhydrite	0.417	0.259	0.017	10.597
		4793	Salt	0.572	0.241	0.04	10.657
12.25	5.875	5263	Anhydrite	0.5	0.704	0.07	12.442
		5544	Shale	0.562	1.047	0.525	17.514
		6013	Shale	0.514	0.659	0.155	13.572
		6575	Sandstone	0.621	0.715	0.288	15.156
		7044	Salt	0.588	0.76	0.333	16.291
		7513	Salt	0.681	0.691	0.265	15.636
		8069	Salt	0.632	0.804	0.279	15.784
		8537	Salt	0.648	0.916	0.282	15.571
		9006	Salt	0.77	0.581	0.322	14.593
		9605	Salt	0.766	0.185	0.193	14.118
		10,167	Salt	0.768	0.762	0.274	14.5
		10,542	Salt	0.763	0.313	0.264	14.453
		11,009	Salt	0.762	0.306	0.181	14.141
		11,571	Salt	0.766	0.294	0.149	14.162
		11,944	Anhydrite	0.539	0.94	0.046	14.028
		12,507	Salt	0.751	0.236	0.132	14.089
		12,831	Salt	0.744	0.245	0.129	14.076
8.5	4.5	12,950	Anhydrite	0.365	1.174	0.097	10.991
		13,200	Sandstone	0.442	0.208	0.162	11.541
		13,450	Sandstone	0.588	0.402	0.079	10.496
		13,500	Limestone	0.578	0.362	0.109	10.93
		13,700	Sandstone	0.541	1.298	0.043	10.045
		13,800	Shale	0.501	1.316	0.052	10.201

In validation with well-x, RBFN model performance is evaluated using the same two metrics (R and AAE) and tested under different values of ε= 0, 0.4, and 0.8. The results indicate that the RBFN model has high accuracy, as evidenced by the low AAE and high R values, which indicate small errors and good alignment between predicted and actual CA values.

These results from the development phase are very similar to this validation phase, indicating good generalization. The consistency between the development phase and the validation phase performance indicates that the model is not overfitting the training data, but rather generalizes well to unseen data, such as the validation well-x dataset. This makes the RBFN model robust and suitable for real-world applications, as it maintains high prediction accuracy even when tested on new datapoints not seen during training.

**Fig. 12:**
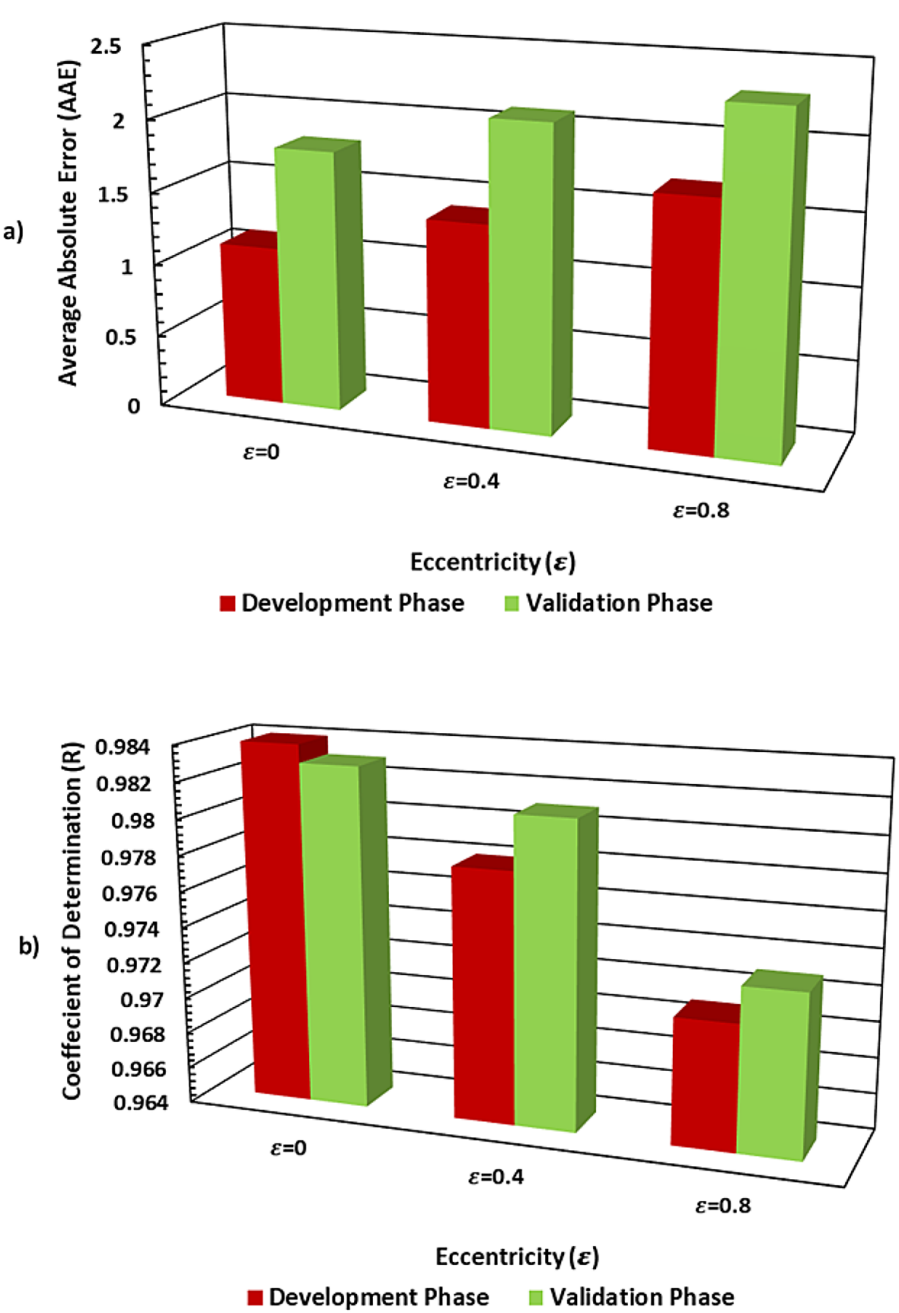
Statistical performance of the development and validation phases of RBFN models: (**a**) R values; (**b**) AAE values.

Same as in the development phase, a slight decrease in R values for ε= 0.8 (0.973 in validation) and (0.971 in development) compared to the other levels can be attributed to the increased complexity in the linear system caused by higher pipe hole eccentricity level increasing (Fig. 12). As eccentricity increases, the behavior of the system becomes more complex, which badly affects the RBFN model ability to generalize effectively. However, even with this drop, the model performance is still strong, indicating that it is able to handle a range of eccentricity values reasonably well.

Moreover, RBFN model exhibited consistently high performance across all cases, with R ranging from 0.988 to 0.996, and R^2^ values above 0.976, confirming strong predictive capability. Adjusted R^2^ values further confirmed the robustness of the model with minimal risk of overfitting. Moreover, the model achieved low RMSE, as low as 0.001, and MAPE under 6%, highlighting its accuracy and reliability. These results validate the generalizability of the RBFN model across varying operational conditions.

**Fig. 13:**
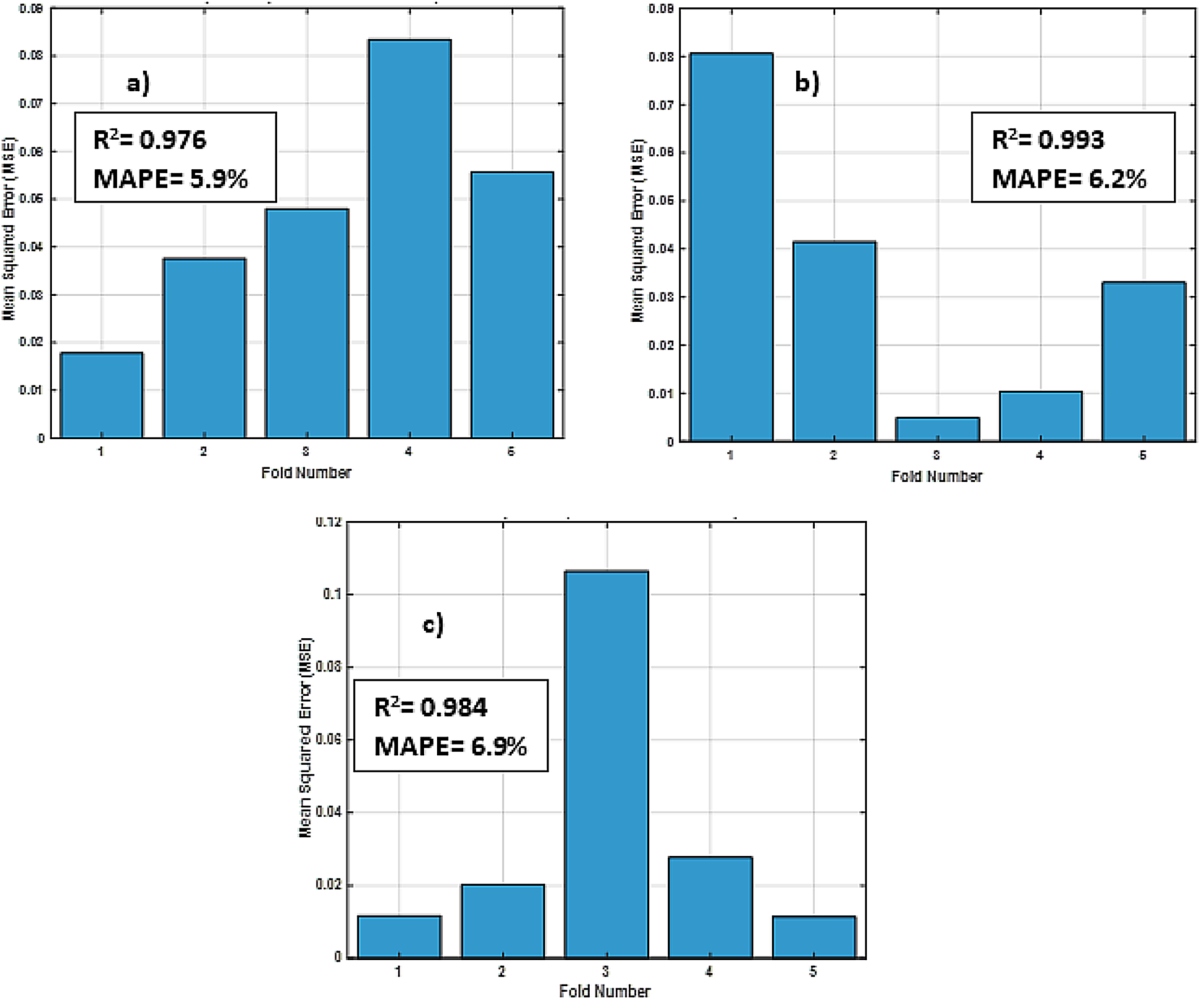
Bar charts of mean squared error (MSE) per fold of: (**a**) ε=0; (**b**) ε=0.4; (**c**) ε=0.8 for RBFN 5-fold cross-validation.

**Fig. 14:**
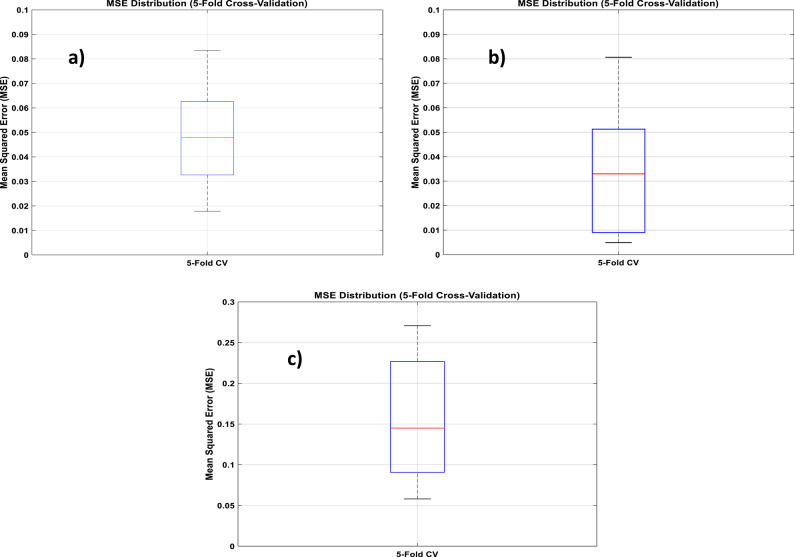
Box plots of mean squared error (MSE) per fold of: (**a**) ε=0; (**b**) ε=0.4; (**c**) ε=0.8 for RBFN 5-fold cross-validation.

**Fig. 15:**
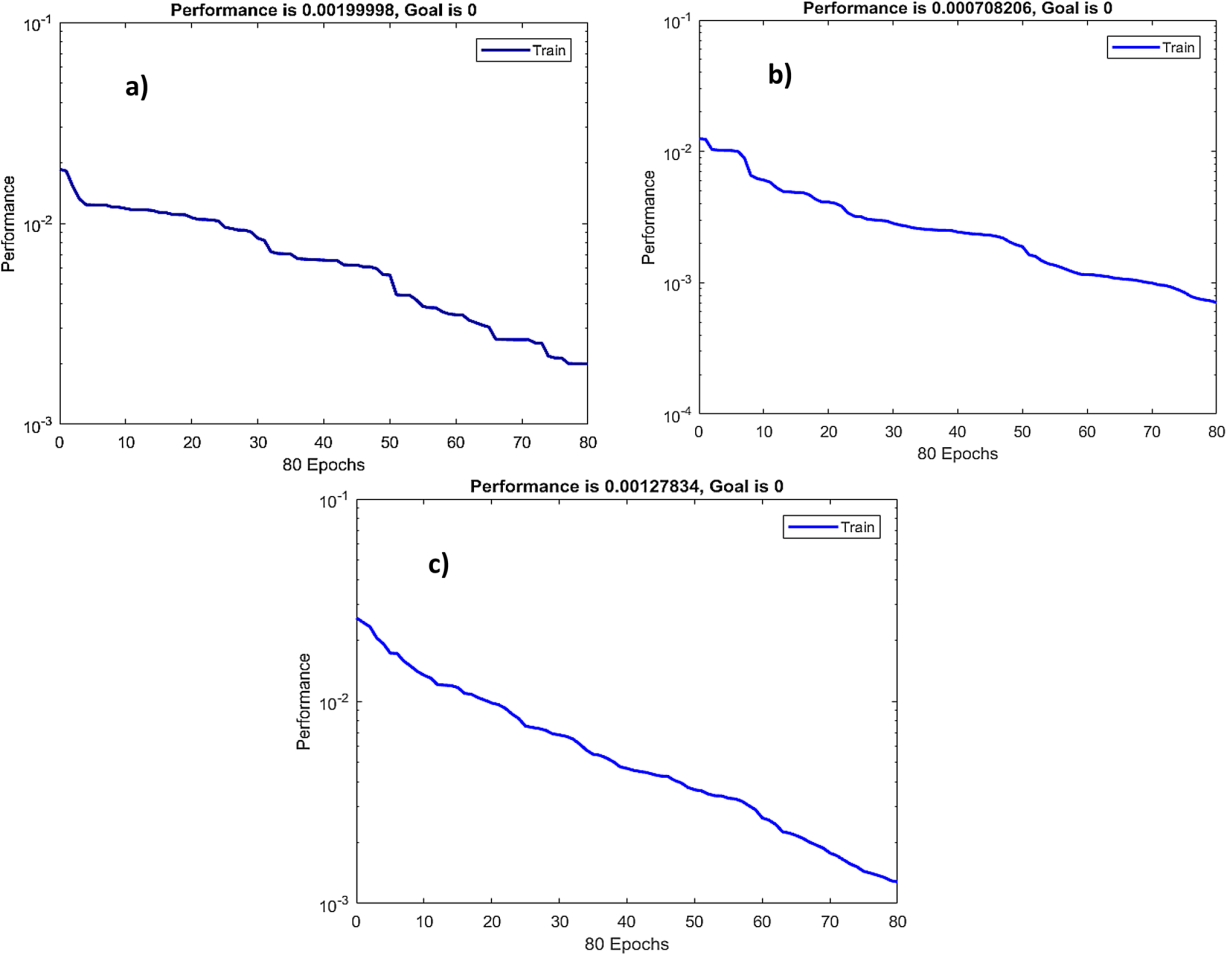
Performance curves RBFN 5-fold cross-validation of: (**a**) ε=0; (**b**) ε=0.4; (**c**) ε=0.8.

A detailed visualization of RBFN model performance presented in (Figs. 13, 14 and 15) showing bar charts of MSE per fold, boxplots of MSE distribution and performance curves across an overall validation dataset, respectively. Additionally, various statistical metrics to measure cross validation performance for each eccentricity level are summarized (Tables 10, 11 and 12), illustrating that no single fold exhibited significant degradation in predictive accuracy. This supports the reliability and robustness of the model under different data partitions.

The observed improvement over initial validation using a static (70, 15, 15%) data split underscores the value of k-fold cross-validation, particularly when dealing with datasets derived from limited operational diversity (six wells from a single basin). The enhanced stability and accuracy of the model reflect effective mitigation of overfitting and improved confidence in the real-world applicability of proposed RBFN model.

**Table 10:** Statistical metrics of (5-fold cross-validation) for (ε=0).

	ε=0							
	R	R^2^		Adjusted. R^2^		AAE	RMSE	MAPE (%)
Overall	0.988	0.976		0.975		1.2	0.021	5.9

**Table 11:** Statistical metrics of (5-fold cross-validation) for (ε=0.4).

	ε=0.4							
	R	R^2^		Adjusted. R^2^		AAE	RMSE	MAPE (%)
Overall	0.996	0.993		0.975		0.6	0.001	6.2

**Table 12:** Statistical metrics of (5-fold cross-validation) for (ε=0.8).

	ε=0.8							
	R	R^2^		Adjusted. R^2^		AAE	RMSE	MAPE (%)
Overall	0.992	0.984		0.992		0.6	0.001	6.9

### Feature importance and sensitivity for proposed RBFN model

**Fig. 16:**
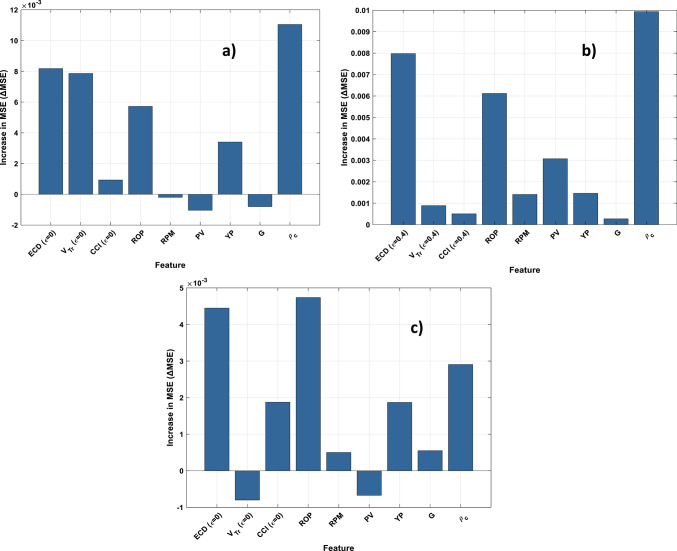
Permutation feature importance (PFI) on test set: (**a**) ε=0; (**b**) ε=0.4; (**c**) ε=0.8 for RBFN modelling.

To assess feature relevance and enhance model interpretability, a Permutation Feature Importance (PFI) analysis was conducted on the test dataset using the final trained RBF neural network (Fig. 16). This method estimates the contribution of each input feature by measuring the increase in prediction error (ΔMSE) after randomly permuting that features values, thereby disrupting its relationship with the target. This approach is well-suited for complex, nonlinear systems such as eccentric deviated hole drilling. PFI was evaluated for three pipe eccentricity conditions ε= (0, 0.4, 0.8) to understand how the influence of each input feature evolves with increasing eccentricity.

Across all cases, ECD remained the most important feature, with ΔMSE values ranging from (0.0044–0.0111), confirming its dominant role in predicting CA regardless of annular geometry. ROP and ρ_c_ were consistently ranked among the top three influential features in all conditions, with ΔMSE values around (0.0038–0.0047) and (0.0022–0.0042), respectively.

The increasing importance of features such as CCI and G with higher eccentricity levels suggests condition-specific interactions, where their influence becomes more pronounced under asymmetric or inclined flow conditions. This indicates that geometric and cuttings transport effects are more critical in deviated wells, and their inclusion in predictive models should be emphasized when modeling non-vertical or eccentric drilling environments.

To examine the statistical behavior and robustness of the proposed RBFN model across different eccentricity configurations, a Monte Carlo simulation was conducted. A total of 10,000 realizations were generated to evaluate the distribution characteristics of the predicted cuttings concentration (CA) at eccentricity levels (=0, 0.4 and 0.8). The simulation process was constructed by randomly sampling input variables from the normalized dataset using stratified random techniques to ensure representation of the full variability in the input space. These samples were then propagated through the trained RBFN model, and the outputs were recorded for statistical evaluation. Such simulation techniques have been previously validated in the literature for approximating the behavior of complex nonlinear systems where analytical solutions are challenging to derive^59^.

**Fig. 17:**
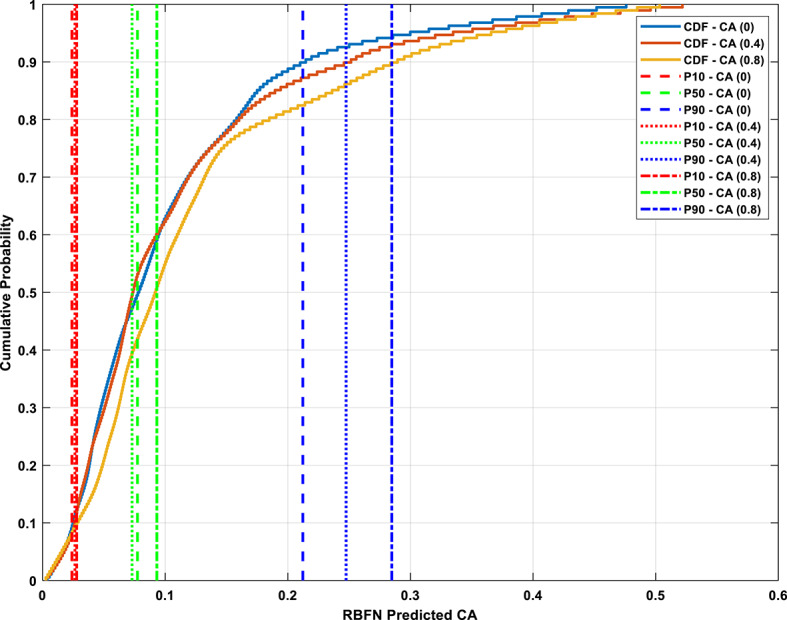
CDF plot of RBFN predicted CA with P10, P50, and P90 percentiles of (ε=0, 0.4 and 0.8) datasets.

Following this, the cumulative distribution functions (CDFs) of the CA predictions were constructed for each eccentricity scenario. Three key percentiles—the 10th (P10), 50th (P50), and 90th (P90)—were calculated to characterize the spread, central tendency, and skewness of each output distribution. These percentiles were found to be aligned with theoretical expectations, where P50 represented the median and P10/P90 provided insight into the tail distribution of the model output. As illustrated in Fig. 17, the resulting CDFs for CA(0), CA(0.4), and CA(0.8) exhibit sigmoidal (S-curve) behavior, with most values concentrated around the median and relatively few extreme values at the tails.

At ε = 0, the CDF curve remains shallow, indicating a wider distribution with most predicted CA values clustered toward the lower end of the scale. As the eccentricity increases to ε = 0.4, the CDF becomes steeper and shifts to the right, representing an increased concentration of mid-range CA values and higher model confidence. This trend is further amplified at ε = 0.8, where the CDF curve is even steeper and levels off more rapidly, signifying that the model outputs are now tightly clustered at higher CA values. This progression confirms the model’s increasing predictive stability with greater eccentricity, as fewer outliers and a tighter output range are observed. Additionally, the upward shift in the P50 value from CA(0) to CA(0.8) quantitatively confirms the anticipated increase in CA with eccentricity, further validating the physical realism of the RBFN predictions.

**Fig. 18:**
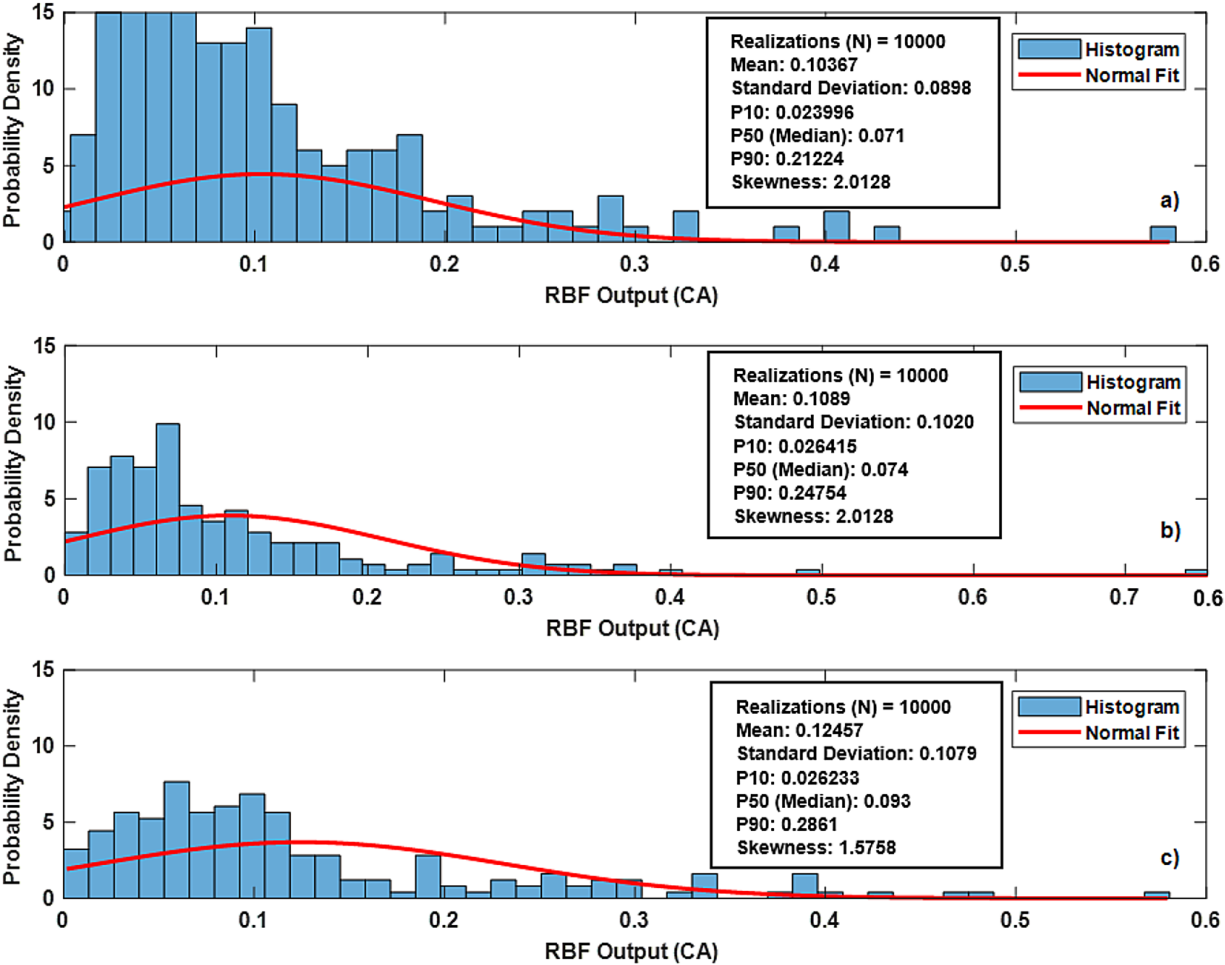
Histogram of Monte Carlo simulated RBFN output (CA) sample with normal distribution fit of (ε=0, 0.4 and 0.8) datasets.

The histogram of the simulated random data, normalized to a probability density function (PDF), revealed a close alignment with the expected normal distribution, confirming the validity of the assumption of normality. In the CA (0) histogram (Fig. 18a), high probability is concentrated at lower values near the left tail, which suggests that the system consistently produces low output values. The distribution is narrow and sharp, with probability peaks close to the lower end of the CA range. This means the RBFN output at low concentrations is highly stable and predictable. There is minimal variation, as most of the output values fall near the mean, indicating a high degree of certainty in the response at this level. For CA (0.4) (Fig. 18b), the probability for low CA decreases compared to CA (0). As the eccentricity level increases, the normal distribution fit (red curve) shifts towards the middle range, and the output values are more likely to fall closer to the mean of the medium concentration range, with less probability for lower CAs. Finally, for CA (0.8) (Fig. 18c), a good normal fit for high concentrations suggests that the high CAs predictions are controlled or have reached an equilibrium, with low variations and a tight clustering around the mean. Further, skewness approach minimal value (1.5758) typical for a normal distribution, indicating fewer extreme deviations and a bell-shaped curve.

Overall, across sensitivity analysis, we can conclude that data exhibit predictable, and well-controlled behavior with minimal noise or skewness, leading to a reliable analysis and high confidence in CA predictions. These findings align with previous sensitivity analysis in the field, where similar Monte Carlo approaches have been used to model and analyze random processes, providing valuable insights into the underlying distributions of predicted CA using proposed RBFN approach.

### Effect of intermediate features on model accuracy

**Fig. 19:**
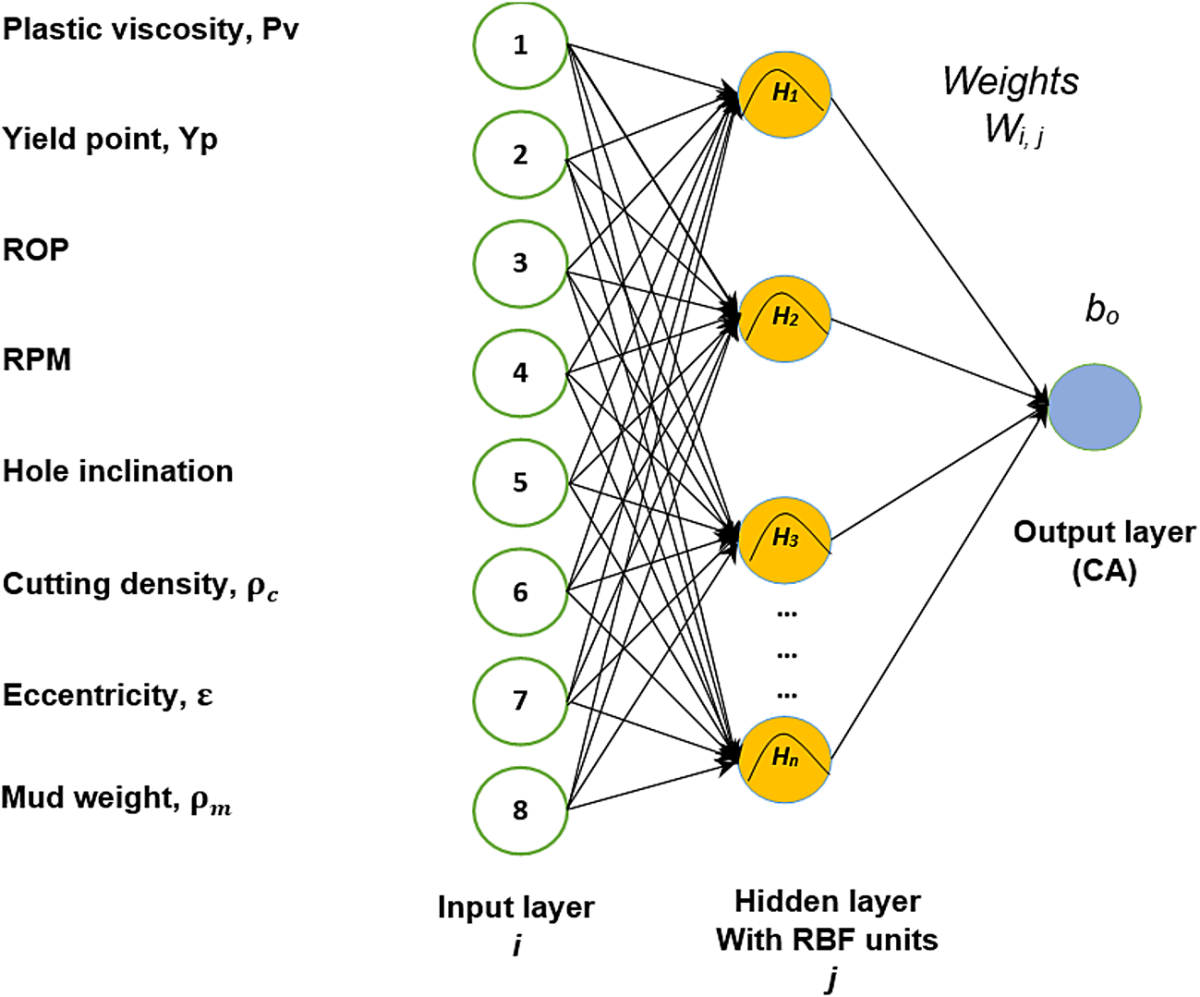
Architecture of RBFN model using raw data for CA prediction.

**Fig. 20:**
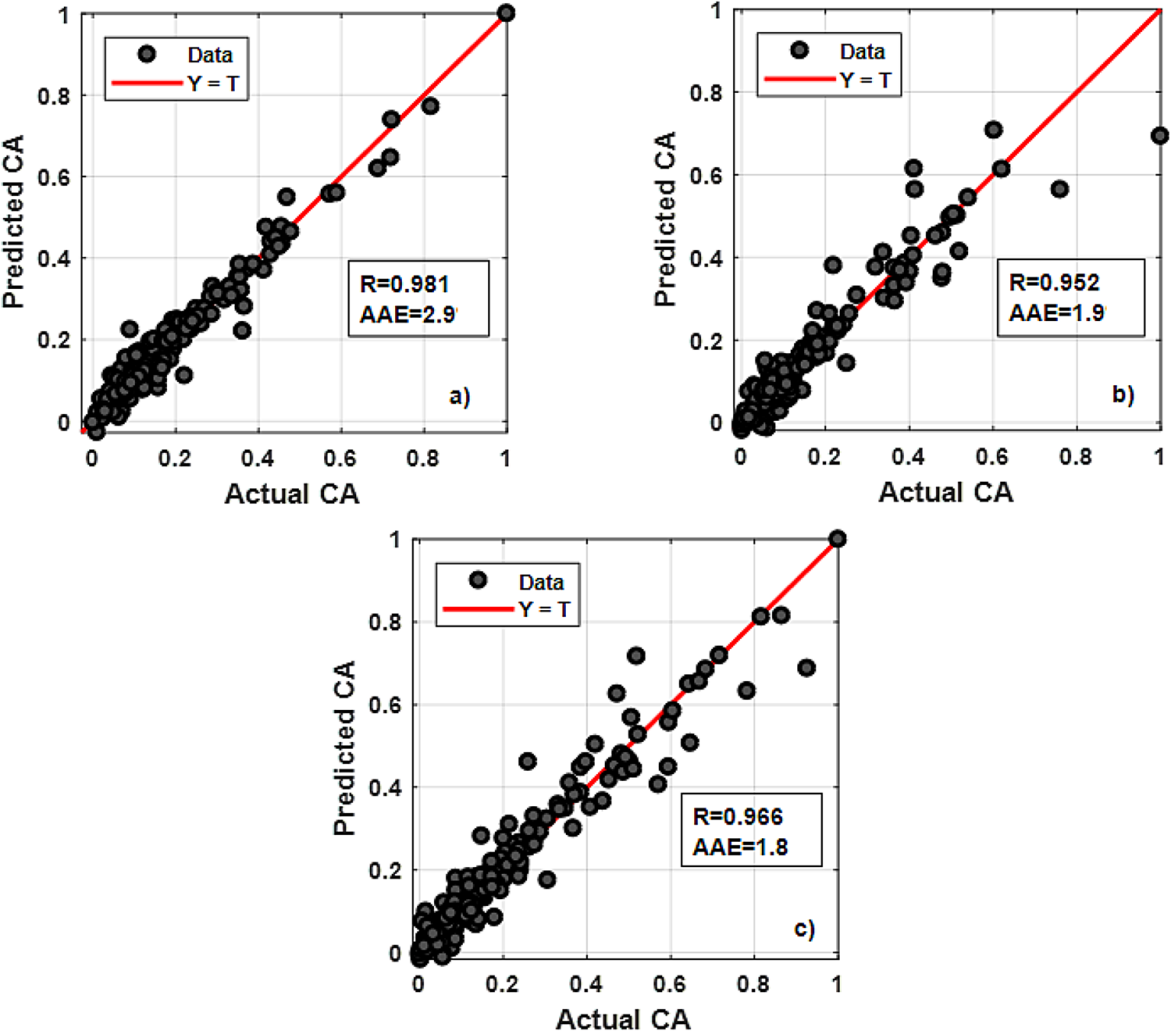
Regression plots of RBFN modelling using raw features for: (**a**) ε=0; (**b**) ε=0.4; (**c**) ε=0.8.

We evaluated the performance of the RBFN model trained with raw features (Fig. 19), such as eccentricity level and $\:{\:\rho\:}_{m}$, compared to the intermediate feature model. The model using raw features performed with overall R and AAE values of (0.981, 0.952 and 0.966) and 2.9, 1.9 and 1.8, for the three presumed levels, respectively (Fig. 20). This indicates that while the model using raw features had a strong performance, the results were not as optimal as those from the model incorporating intermediate features.

The intermediate features significantly enhanced the predictive power of the RBFN model, as shown by the higher R and lower AAE values. This highlights the importance of using well-chosen intermediate variables to improve the predictive model accuracy and CA prediction capabilities, especially in complex eccentric geometries where raw features alone may not fully capture the required patterns. The model incorporating intermediate variables (V_TR_, CCI, ECD) achieved an RMSE of 8.7, demonstrating a significant improvement in predictive accuracy.

### White box equations based on developed RBFN model

RBF output function can be formulated in terms of Euclidian distance coefficient $\:{\text{h}}_{\text{i}}\left(\text{x}\right)$ Gaussian basis function as:

26 $$\:{y}_{n}=\sum\:_{i=1}^{m}{w}_{i,n}{h}_{i}\left({x}_{n}\right)\:+{b}_{n},$$

where;

27 $$\:{h}_{i}\left({x}_{n}\right)={e}^{\left(\frac{-{(x-{c}_{i})}^{2}}{{{r}_{i}}^{2}}\right)},$$

Equation 18 can be solved by solving a system of linear equations using least squares method as:

$$\:{[h}_{1}\left({x}_{1}\right){w}_{1}+{h}_{2}\left({x}_{1}\right){w}_{2}+\dots\:+\:{h}_{9}\left({x}_{1}\right){w}_{9}]+{b}_{1}={y}_{1}\:\:\:\:$$

$$\:\left[{h}_{1}\left({x}_{2}\right){w}_{1}+{h}_{2}\left({x}_{2}\right){w}_{2}+\dots\:+\:{h}_{9}\left({x}_{2}\right){w}_{9}\right]+{b}_{2}={y}_{2}\:\:\:\:$$

$$\::=\dots\:.,\:$$

$$\::=\dots\:.,\:\:$$

28 $$\:\left[{h}_{1}\left({x}_{n}\right){w}_{1}+{h}_{2}\left({x}_{n}\right){w}_{2}+\dots\:+{h}_{9}\left({x}_{n}\right){w}_{9}\right]+{b}_{n}={y}_{n},$$

Here, the design matrix W*H + b = y includes H coefficients on the left-hand side of the equation, the weight vector W added by the output layer bias b to produce the optimized output function y. The entire mathematical operation is repeated for every RBFN model for different eccentricities (0, 0.4, 0.8). An excerpt of RBFN centers (C) and coefficients (H) for each eccentricity model are listed in (Table 13).

**Table 13:** An excerpt of the developed RBF centers and H coefficients based on 4512 field observations.

**RBF centers:** C = 0.2620 0.2829 0.2829 0.2829 0.2833 0.2833 0.2822 0.2822 0.2822 0.2836 0.2836 0.2836 0.3613 12.1000 12.1000 12.1000 12.2000 12.3000 12.4000 12.4500 12.4000 12.4000 12.5000 12.6000 12.6000 12.6000 23.2000 40.8000 40.8000 40.8000 40.8000 30.8000 30.8000 32.2000 32.2000 32.2000 28.3000 28.3000 60.3000 180.0000 250.0000 250.0000 250.0000 250.0000 250.0000 250.0000 250.0000 250.0000 250.0000 251.0000 250.0000 250.0000 25.0000 30.0000 30.0000 30.0000 31.0000 31.0000 33.0000 33.0000 33.0000 32.0000 32.0000 32.0000 39.0000 42.0000 36.0000 36.0000 36.0000 37.0000 37.0000 40.0000 40.0000 40.0000 38.0000 38.0000 38.0000 23.0000 22.9625 17.9525 24.8830 22.9625 17.9525 17.9525 24.8830 24.8830 22.9625 24.8830 22.9625 24.8830 24.8830 1.3787 1.3791 1.3819 1.3777 1.3723 1.3719 1.3733 1.3731 1.3731 1.3733 1.3744 1.3731 1.3728 …….
**RBF H coefficients**: H = 0.0699 0.0741 0.0720 0.0698 0.0710 0.0695 0.0705 0.0694 0.0696 0.0699 0.0741 0.0720 0.0698 0.0710 0.0695 0.0705 0.0694 0.0696 0.0699 0.0741 0.0720 0.0698 0.0710 0.0695 0.0705 0.0694 0.0696 0.0699 0.0715 0.0753 0.0706 0.0703 0.0695 0.0706 0.0695 0.0694 0.0701 0.0709 0.0754 0.0710 0.0714 0.0695 0.0703 0.0707 0.0694 0.0699 0.0725 0.0732 0.0705 0.0715 0.0695 0.0706 0.0694 0.0694 0.0701 0.0709 0.0757 0.0710 0.0720 0.0695 0.0703 0.0696 0.0694 0.0699 0.0709 0.0785 0.0703 0.0713 0.0695 0.0703 0.0694 0.0694 0.0699 0.0717 0.0848 0.0702 0.0711 0.0695 0.0706 0.0694 0.0694 0.0699 0.0707 0.0772 0.0710 0.0705 0.0695 0.0706 0.0695 0.0694

## Comparison between ML and CA empirical models

### Empirical models description

The first comparison will be conducted with^34^ model. They conducted an experimental investigation that was to assess the effectiveness of mechanical cleaning devices (MCD) in removing cuttings and their applicability for improving extended reach drilling (ERD) systems. A large-scale flow loop with an 8-inch transparent test segment was used for the experiments. The analysis considered three distinct drill pipe diameters. They incorporated the most important factors, including flow rate, ROP, diameter ratio, tool design, and drill pipe rotation speed. Fewer tests were conducted using the Taguchi approach by employing a statistical technique with orthogonal arrays in the experiment design to examine many samples with fewer trials. Also, they used weight measurements to calculate CA that had accumulated in the test portion for each test.

Their findings indicate that the inclination angle and flow rate are the most important factors. In conclusion, they developed correlations that help create an analytical model to optimize drilling systems using MCDs, either in terms of drill string design or operational parameters.

The second model^23^ considered in this paper is based on developing a computational intelligence FL technique to develop a model for estimating CA. Their work proved that FL could handle data fuzziness and simulate non-linear functions with arbitrary complexity, much like human thinking. Further, they compared the FL model estimations they produced for CA. Their work is based on 509 experimental data with 11 separate test parameters each. Further, they compared their produced FL model estimations of CA with the two empirical models proposed by Chowdhury and Skalle (2017) for a test dataset consisting of nine experimental observations gathered from^34^.

Ref^35^. developed the third empirical model that was taken into consideration for comparison in this work. In order to create a trustworthy CA prediction tool, oil field data from six offshore deviated wells was used to build the model, which was based on the Buckingham Pi theorem. For three different and presumed eccentricity values (ε= 0, ε= 0.4 and ε= 0.8), a least linear regression approach was used, considering seven dimensionless groups of drilling parameters, rheological parameters, cuttings density, and indicators of hole cleaning efficiency such as equivalent circulating density, cutting transport velocity ratio, and carrying capacity index.

Moreover, empirical models exclusively assume power-law rheological models to account for the annuli velocity profiles that were demonstrated. Additionally, to quantify the influence of eccentricity on hole cleaning, this study relies only on the assumption of steady state flow of non-Newtonian fluids. In their regression models, only the values of ε= 0, ε= 0.4, and ε= 0.8 were assumed. For the other eccentricity scenarios, CA will thus be 0 and independent of the drilling parameters. Further, they developed CA model is only applicable on wells that are deviated up to about 62º degrees of inclination.

The comparison models are referred to as “*Mod* 1”, “*Mod* 2” and “*Mod* 3”, respectively. These three models were chosen because they are grounded in two distinct modelling methodologies, namely dimensional analysis and FL intelligence. Therefore, it is necessary to evaluate their CA estimation accuracy with other ML algorithms employed in the current work. The description and drawbacks of *Mod* 1, 2 and 3 are listed in (Table 14).

**Table 14:** The comparison models.

Mod 1
$\:CA=\left(1-\varnothing\:\right){A}_{c}$ $\:{A}_{c}={A}_{0}{\varnothing\:}^{{A}_{1}}{S}_{T}^{{A}_{2}}{Re}^{{A}_{3}}{C}_{d}^{{A}_{4}}$ $\:{A}_{0}=2.55\times\:{10}^{8},\:{A}_{1}=1.94,\:{A}_{2}=-0.842,\:{A}_{3}=-2.22,\:{A}_{4}=0.174$ $\:{A}_{4}=\left(1-\varnothing\:\right){A}_{c}$ where $\:\text{C}\text{A}$ is cuttings concentration (%), ∅ is the cuttings bed porosity$\:,\:{A}_{c}\:$is the ratio of cuttings bed to annulus cross-sectional area (%)$\:,\varnothing\:$ is the inclination angle (radians), $\:{S}_{T}$ is the Strouhal number, $\:\text{R}\text{e}$ is the Reynolds number, $\:{\text{C}}_{\text{d}}$ is the delivery concentration, $\:\left({A}_{0}-{A}_{4}\right)$are constants.
**Limitations** • The condition in which θ = 0 is not included in the model. In this instance, CA will be zero regardless of the values of the other drilling parameters, such as flow rate, ROP, or RPM. • This model does not consider pipe hole eccentricity. • The model does not include the no drill string rotation condition.

Mod 1
FL developed rules have the following generic structure: $\:IF\:\left(Ann\:is\:A\right)and\:\left(Incl\:is\:B\right)and\:\left(Ecc\:is\:C\right)and\:\left(Temp\:is\:D\right)and\:\left(Rhof\:is\:E\right)and\:\left(Vapp\:is\:F\right)and\:\left(Cuts\:is\:G\right)and\:\left(Rhos\:is\:H\right)$ $\:and\:\left(RPM\:is\:I\right)\:and\:\left(Qf\:is\:J\right)\:and\:\left(ROP\:is\:K\right)\:then\:CA\:is\:L\:\left(weight\right)\:\:$ where Rhof is drilling fluid density, Vapp is the apparent viscosity, Ann is the annulus size, Ecc is the eccentricity, $\:Incl$ is angle of inclination, Temp is temperature, Qf is pump rate, RPM is the drill string rotation, penetration rate (ROP), (A – L) stand for linguistic values, and weight denotes a value between (0.5, 0.75, and 1.0).
**Limitations** • FL model does not incorporate artificial neural network and genetic algorithm for CA prediction and need more training data for prediction accuracy.

Mod 3
$\:{CA}_{\:{\upepsilon\:}=0}=0.66{\left(\frac{Yp}{{ROP}^{2\:}\:\text{*}{\rho\:}_{c}}\right)}^{\left(-0.6\right)}{\left(\frac{{\rho\:}_{c}}{\:ECD}\right)}^{\left(-0.77\right)}{\left(G\right)}^{\left(-0.42\right)}{\left(\frac{Pv\text{*}RPM}{Yp}\right)}^{\left(0.12\right)}{\left({V}_{TR}\right)}^{\left(-2.16\right)}\:{\left(CCI\right)}^{\left(0.36\right)}$ $\:{CA}_{\:\epsilon\:=0.4}=0.1{\left(\frac{Yp}{{ROP}^{2\:}\:\text{*}{\rho\:}_{c}}\right)}^{\left(-0.77\right)}{\left(\frac{{\rho\:}_{c}}{\:ECD}\right)}^{\left(-2.53\right)}{\left(G\right)}^{\left(1.81\right)}\:{\left(\frac{Pv\text{*}RPM}{Yp}\right)}^{\left(-0.90\right)}\:{\left({V}_{TR}\right)}^{\left(-4.5\right)}{\left(CCI\right)}^{\left(0.63\right)}$ $\:{CA}_{\:{\upepsilon\:}=0.8}=0.007{\left(\frac{Yp}{{ROP}^{2\:}\:\text{*}{\rho\:}_{c}}\right)}^{\left(-0.52\right)}{\left(\frac{{\rho\:}_{c}}{\:ECD}\right)}^{\left(-2.51\right)}{\left(G\right)}^{\left(1.26\right)}{\left(\frac{Pv\text{*}RPM}{Yp}\right)}^{\left(-0.26\right)}\:{\left({V}_{TR}\right)}^{\left(-2.61\right)}{\left(CCI\right)}^{\left(-0.22\right)}$ where $\:\text{C}\text{A}$ is cuttings concentration,$\:\text{Y}\text{p}\:$ is the yield point, $\:{\rho\:}_{c}$ is the cutting density, ECD is the equivalent circulating density, $\:\text{P}\text{v}$ is the plastic viscosity, $\:\text{R}\text{P}\text{M}$ is the drill pipe rotation, $\:{\text{V}}_{\text{T}\text{R}}$ is the cutting transport velocity ratio, $\:\text{C}\text{C}\text{I}$ is the carrying capacity index.
**Limitations** • The estimation of the ε effect on hole cleaning in this work is based only on the assumption of steady state flow of non-Newtonian fluids. • The only assumptions made by the regression model were that ε = 0, ε = 0.4, and ε = 0.8. Therefore, regardless of the drilling settings, CA will be 0 for the other eccentricity situations. • The developed model only accounts for wells with 62º degrees of inclination.

### Comparison results

The proposed best evaluation of the RBFN model was compared with the ML models developed here in this work in addition to the aforementioned empirical models. Noting, all the comparison models had the applicability of studying the eccentricity variation effect on CA prediction except for *Mod* 1, which has a shortcoming of eccentricity as a variated variable in their developed empirical correlation. So, the evaluation between all models will be limited to the application of pipe hole eccentricity level of (= 0.5) for comparison.

Table 15 lists the evaluation matrices R and AAE that are used to compare the CA estimates made by ML models and the three comparison models for the test dataset comprising nine observations adopted by *Mod* 2 and originating from a published dataset of *Mod* 1 (Table 16). The average values of the test dataset used for comparison between models and used in Mod 2 are listed in (Table 17).

**Table 15:** Comparison results of developed RBFN model.

	ε=0.5	
	*R*	AAE
RBFN model	0.972	1.25
BPNN model	0.815	5.41
SVM model	0.924	1.21
^34^ empirical correlation	0.823	2.43
^23^ model	0.921	2.34
^35^ statistical model	0.843	2.41

**Table 16:** An overview of the experimental data utilized in *Mod* 1 experimental setup and adopted by *Mod* 2.

Mod 1	Annulus Size (in)	Eccentricity	ROP (ft/min)	Temperature (º C)	Test Pressure (psi)	RPM	Inclination (◦)	Cuttings Size (in)	Cuttings Packed Porosity	Cuttings Density (ppg)
^34^	4.5	+ 0.5	0.7–1.3	27	14.5	90–140	40–90	0.13	0.4	21.7
	3.5									
	3.0									
	25.9									

**Table 17:** Linguistic ranges of experimental test data that formulated by mod 2 and originated from mod 1.

Test variable	Input ranges
Annulus size (in)	0–5
Inclination (◦)	0–60
Eccentricity	0–0.5
Temperature (º C)	0–82
Drilling fluid density (ppg)	0–14
Cuttings size (in)	0–0.25
Apparent viscosity (cp.)	1–251
Cuttings density (ppg)	0–22.3
RPM	0–150
Drilling fluid flow rate (gpm)	0–4.16
ROP (ft/hr)	0–18.4
Cuttings concentration CA (%)	0–50%
V_TR_	0.21–0.63
CCI	0.4–1.06
ECD (ppg)	10.2–14.7

The results demonstrate that the developed RBFN was much more effective in this work than the previously reported CA comparison models and other utilized ML algorithms in terms of AAE and R evaluation metrics.

### Real-time applicability and implementation feasibility

The developed RBFN-based prediction framework demonstrates strong potential for real-time field deployment. Once trained, the RBF network performs predictions with minimal computational overhead, typically requiring only matrix multiplications, making it highly suitable for integration with real-time drilling monitoring systems. The inference speed is near-instantaneous (< 1 ms per prediction on a standard CPU), which satisfies the time constraints of real-time decision-making in drilling operations.

Computational Cost during the training phase is modest. For example, training the final RBFN with up to 32 neurons on a dataset of ~ 1200 data samples complete in under 30 s on a standard laptop (Intel i7, 16 GB RAM). More advanced models like BPNN and SVM, though slightly more intensive, still remain practical for deployment on typical rig-side or remote servers.

To address model adaptability, a retraining strategy can be employed using either: rolling updates (e.g., retrain weekly with recent data from the well site), or online learning for certain models (e.g., adaptive SVM variants). Accordingly, these strategies ensure that the model remains responsive to changing formation conditions, tool wear, or fluid properties. Future deployment may also benefit from edge computing devices or cloud-based retraining pipelines that synchronize with rig data acquisition systems.

This approach supports scalable, maintainable, and field-ready implementation of ML-driven hole cleaning optimization in deviated well drilling.

## Conclusions

This study developed various machine learning (ML) models to predict cutting concentration (CA) and optimize hole cleaning efficiency in deviated well drilling, using field data from six inclined wells under different eccentricity () degrees. These are the main findings:

- The radial basis function (RBFN) algorithm was successfully applied for real-time CA prediction, demonstrating superior performance over existing empirical models.
- Incorporating intermediate features — cutting transport velocity ratio (V_TR_), carrying capacity index (CCI), and equivalent circulating density (ECD) — into the RBFN model significantly improved predictive accuracy in terms of absolute average error (AAE) and correlation coefficient (R), compared to models using only raw input features. This highlights the value of using domain-derived intermediate variables for more accurate predictions.
- Validated RBFN models exhibited high accuracy in a neighboring test well, achieving AAE-values of 1.8, 2.1, and 2.3, and R values of 0.983, 0.981, and 0.973 for = 0, 0.4, and 0.8, respectively.
- The permutation feature importance analysis revealed that while features like ECD, ROP, and cutting density (ρ_c_) consistently drive model predictions, the rising influence of CCI and geometric factor (G) at higher eccentricities highlights the need for condition-specific modeling. These findings emphasize that in eccentric or inclined drilling scenarios, incorporating transport-related and geometric parameters is essential for accurate, reliable prediction of cutting concentration.
- Monte Carlo simulation with 10,000 realizations confirmed that the RBFN model outputs are statistically stable and normally distributed, with strong predictability and minimal skewness at high eccentricity levels.

The application of these ML models represents a novel approach to enhancing operational efficiency in deviated drilling, offering significant advancements in real-time drilling analytics.

The model’s rapid inference speed and low computational demand make it well-suited for real-time deployment at the rig site, particularly for monitoring hole cleaning efficiency under varying eccentricity conditions. Additionally, the study underscored the importance of integrating advanced ML models like BPNN and SVM to refine predictive accuracy under variable eccentricities, specifically ε= 0.5.

### Recommendations for future potential

Conclusions of this work highlight the necessity of employing field datasets with ML models to estimate CA of different eccentric and deviated annuli using readily available field values as input variables. Nevertheless, this ML regression model has several limits in terms of its application:

- Estimating the ε effect on hole cleaning in this work is based only on the assumption of steady state flow of non-Newtonian fluids.
- For all deviated wells taken into consideration in this study, the measured values of CA are at ε= 0, which is the basic scenario. The only eccentricity assumptions made by ML regression models were ε= (0, 0.4, and 0.8).
- Only deviated wells up to about 62º degrees of inclination that can be used with developed intelligent CA models, so conducted comparison in this work is limited to inclinations (0–60º) in the given test dataset.
- Although this study focused on RBF neural networks for their balance of accuracy and interpretability, future work may benefit from benchmarking against ensemble models such as Random Forest and Gradient Boosted Trees to further assess performance trade-offs in predictive drilling applications.
- Future work could explore hybrid models that combine data-driven learning with physical constraints or empirical knowledge. Such approaches may improve generalization and ensure predictions remain consistent with known drilling principles.

Therefore, to evaluate the eccentricity influence on CA and hole cleaning efficiency, further study is needed to consider demonstrating annulus velocity profiles slip velocity correlations for the condition of unsteady state flow of power-law fluids. In addition, it is recommended extending the RBFN applicability to more training datasets and different eccentric profiles for predicting CA in highly deviated wells.

## Data Availability

The raw data supporting the conclusions of this article will be made available by the authors on request (contact: Mohamed Y. Saad mfys1@pme.suezuni.edu.eg).

## Abbreviations

$\:({\alpha\:}_{1}-\:{\alpha\:}_{2}$: RBFN fusion weights
$$\:\left({\text{A}}_{0}-{\text{A}}_{4}\right)$$: Correlation constants
$\:{{\varnothing}}_{i}$: Hidden neuron basis function
A_c_: Ratio of cuttings bed to annulus cross sectional area
N_h_: Number of hidden neurons
N_i_: Number of input neurons
Q_1_: First quartile
Q_3_: Third quartile
S_T_: Strouhal number
V_s_: Slip velocity
X_max_: Maximum value of X
X_min_: Minimum value of X
X_n_: Normalized value of X
*H*_*j*_: j-th hidden unit radial basis output
*I*_*j*_: Relative importance of input variable
*b*_*n*_: Output neurons bias
*c*_*i*_: Centers of RBF network
*r*_*i*_: RBFN radii
*w*_*i,n*_: Synaptic weights linking hidden layer to output neurons
*w*_*jk*_: Output layer wight
$$\left\| {x - c_{i} } \right\|$$: Euclidean distance
∅: Cuttings bed porosity
AAE: Average absolute error
ANN: Artificial neural network
BPNN: Back propagation neural network
CA: Cuttings concentration
CCI: Carrying capacity index
V_TR_: Cuttings transport velocity ratio
CDF: Cumulative distribution function
DH: Wellbore diameter
DO: Drill pipe diameter
ECD: Equivalent circulating density
ERD: Extended reach drilling
FL: Fuzzy logic
G: Hole geometrical factor
h: Hidden layer
i: Input layer
IQR: Inter quartile range
LIB: Lower inner boundary
MAPE: Mean absolute percentage error
MCD: Mechanical cleaning devices
ML: Machine learning
Mod: Model
NPT: Non-productive time
n^th^: Learning iteration of particular epoch
o: Output layer
PFI: Permutation feature
Pv: Plastic viscosity
R: Relation coefficient
RB: Radial basis
RBF: Radial basis function
RBFN: Radial basis function network
RMSE: Root mean squared error
ROP: Rate of penetration
RPM: Drill pipe rotation
SVM: Support vector machine
UIB: Upper inner boundary
WOB: Weight on bit
Yp: Yield point
ε: Pipe hole eccentricity
θ: Wellbore inclination
ρ_c_: Cuttings density
$$f\left(\left\| {x - c_{i} } \right\|\right)$$: Gaussian function
